# Genomic basis of the differences between cider and dessert apple varieties

**DOI:** 10.1111/eva.12270

**Published:** 2015-06-13

**Authors:** Diane Leforestier, Elisa Ravon, Hélène Muranty, Amandine Cornille, Christophe Lemaire, Tatiana Giraud, Charles-Eric Durel, Antoine Branca

**Affiliations:** 1 UMR 1345 Institut de Recherche en Horticulture et Semences, Université d’AngersAngers, France; 2 UMR 1345 Institut de Recherche en Horticulture et Semences, INRABeaucouzé, France; 3 Ecologie, Systématique et Evolution, Université Paris-SudOrsay, France; 4 Ecologie, Systématique et Evolution, CNRSOrsay, France

**Keywords:** BayeScan, *F*_ST_, genomewide association, linkage disequilibrium, *Malus domestica*, outlier

## Abstract

Unraveling the genomic processes at play during variety diversification is of fundamental interest for understanding evolution, but also of applied interest in crop science. It can indeed provide knowledge on the genetic bases of traits for crop improvement and germplasm diversity management. Apple is one of the most important fruit crops in temperate regions, having both great economic and cultural values. Sweet dessert apples are used for direct consumption, while bitter cider apples are used to produce cider. Several important traits are known to differentiate the two variety types, in particular fruit size, biennial versus annual fruit bearing, and bitterness, caused by a higher content in polyphenols. Here, we used an Illumina 8k SNP chip on two core collections, of 48 dessert and 48 cider apples, respectively, for identifying genomic regions responsible for the differences between cider and dessert apples. The genome-wide level of genetic differentiation between cider and dessert apples was low, although 17 candidate regions showed signatures of divergent selection, displaying either outlier *F*_ST_ values or significant association with phenotypic traits (bitter versus sweet fruits). These candidate regions encompassed 420 genes involved in a variety of functions and metabolic pathways, including several colocalizations with QTLs for polyphenol compounds.

## Introduction

Domestication and variety diversification have been models for studying the mechanisms underlying adaptation since Darwin (1987), being the result of a strong and recent selection by humans for desired traits in organisms used as food (Meyer et al. 2012; Larson and Burger 2013; McTavish et al. 2013), ornaments (Yuan et al. 2014), pets (Axelsson et al. 2013), or for their metabolic abilities (Douglas and Klaenhammer 2010). Dissecting the genomic changes occurring during domestication and variety diversification has thus a fundamental importance for our understanding of evolutionary processes, in addition to applied interests for improving the desired traits in domesticated organisms and managing the germplasm diversity. Studying the footprints of adaptation in genomes may indeed allow to identify the important traits or metabolic pathways that were under selection during domestication and variety diversification, as well as the genetic bases of these traits (Wang et al. 1999, 2005; Whitt et al. 2002; Palaisa et al. 2003; Gallavotti et al. 2004; Yamasaki et al. 2005; Walsh 2008). Identifying the genomic regions involved may accelerate further improvement of traits controlling agricultural productivity and performance, such as yield, organoleptic or nutritional quality, and resistance to biotic and abiotic stresses, using marker-assisted selection (Soller 1994; Collard and Mackill 2008; Prada 2009). It may also help conservation management programs aiming at maintaining important functional biodiversity in core collections as well as in wild relatives of crop species.

The cultivated apple tree (*Malus domestica* Borkh.) is one of the most important fruit crops in temperate regions, with great economic and cultural values (Juniper and Mabberley 2006). Dessert apples are popular because of their taste, nutritional properties, storability and convenience of use. The fruits of the specific varieties used to produce cider are smaller and bitter, as are those from crabapples, that is, the fruits of the wild apple species. The bitterness is due to a high content in polyphenols (Sanoner et al. 1999). Not all cider cultivars are, however, extremely bitter (Pereira-Lorenzo et al. 2009). Cider apples are also known for their fibrous structure, which allows longer storage (Lea and Piggott 2003; del Campo et al. 2005). In addition, cider apples more often display biennial bearing (Dapena et al. 2005), that is, with crop occurring only every two years. Finally, cider apples are more susceptible than dessert apples to fire blight, a disease caused by the bacteria *Erwinia amylovora* (Paulin et al. 1988; Lespinasse and Paulin 1990). Thousands of apple cultivars have been documented (Morgan et al. 2002), although only a few now dominate the market. Surprisingly, the history of apple domestication has just begun to be unraveled (Cornille et al. 2014). Genetic analyses have revealed a Central Asian origin of cultivated apple, with an initial divergence from the wild species *Malus sieversii*, together with an unexpectedly large secondary contribution through introgression from the European wild species *Malus sylvestris* (Velasco et al. 2010; Cornille et al. 2012). In contrast to expectations, cider cultivars did not appear the most introgressed by wild species based on microsatellites (Cornille et al. 2012). This suggests either a recent selection in the cider varieties for traits favorable for apple-based beverages from the standing genetic variation in the domesticated gene pool, or the introgression of only few genes from crabapples into the cider varieties. However, cider beverage has been produced for centuries in Western Europe especially by the Celts using native crabapples even before the invasion of the Romans who brought the domesticated apples. Much effort has been devoted since the 17th century in Europe to generate cider apple cultivars with high contents in sugar and polyphenols for producing high-quality cider (Morgan et al. 2002).

Although some *M. sieversii* individuals produce large apples, the variability in fruit size and color is wide. The selection by humans in cultivated apples targeted many phenotypic traits, including among others the number of fruits, their size, color, shape, flavor, taste, texture, storage capacity, harvesting ease, juvenile phase length and disease resistance (Janick 2005). QTL mapping has been used to dissect the genetic architecture of several desired traits, through crosses between cultivars (Calenge et al. 2004; Segura et al. 2008; Celton et al. 2011; Guitton et al. 2012; Longhi et al. 2012; Verdu et al. 2014). However, the footprints of selection have been little studied so far in apples compared to annual crops (Yamasaki et al. 2005; Camus-Kulandaivelu et al. 2008). The recently released ‘Golden Delicious’ genome sequence (Velasco et al. 2010) and the availability of medium-density genotyping tools (Chagne et al. 2012a) have made it possible to generate population-scale data for investigating genome-wide patterns of selection.

In this study, we set out to identify genomic regions under divergent selection between cider and dessert apples using two core collections, one of each variety type (*N* = 48 each), and 3704 SNP markers. First, we analyzed the population genetic structure in our sample to assess the differentiation between dessert and cider apple varieties using a much higher number of markers than in a previous study (Cornille et al. 2012). We also investigated the extent of linkage disequilibrium (LD) as a function of genomic distance within the genome to infer the expected maximal distance between the causal variation and the markers displaying association with the phenotype. We then looked at *F*_ST_ statistics for identifying outlier loci that would differentiate cider and dessert varieties significantly more than the average genomic background. Finally, a genome-wide association analysis was performed, taking into account genetic structure and kinship, contrasting the (i) cider versus dessert variety types or (ii) high versus low bitterness cultivars. Altogether, these analyses aimed at localizing the genomic regions that have been under divergent selection and responsible for the phenotypic differences between cider and dessert apples. We then examined in these regions the putative functions of genes to find candidates that have potentially undergone differential changes during the divergence between cider and dessert apples. Recent selection programs on cider apples aim at improving yield, regularity of production, resistance to pests and pathogens, while maintaining their specific technologic characteristics (e.g., high content in polyphenols). The identification of the genomic regions responsible for the differences between dessert and cider variety types could therefore be of great use for instance in a marker-assisted selection approach trying to select new cider varieties combining a higher content in polyphenols with the agronomic performances of dessert apples such as regular annual bearing, higher yield, and fruit size.

## Material and methods

### Plant material

The two apple core collections used in this study had been previously constituted by choosing the individuals that maximized the genetic diversity based on a set of 24 microsatellite markers in the INRA Angers germplasm collection of dessert and cider apple cultivars. Shortly, the core collections were built by retaining individuals from larger sets of apple accessions (737 and 188 for dessert and cider apples, respectively) using the ‘Maximum Length Subtree’ option of the DARwin software (Perrier et al. 2003; Perrier and Jacquemoud-Collet 2006). The two core collections included 48 dessert and 48 cider apple cultivars, respectively (Supporting information). Reflecting the content of the INRA germplasm collection, both core collections mainly include old (generated before the 1950’s) French apple cultivars, some of them being clones of cultivars grown in other European countries under different names. Because Western Europe has been the main place where the selection of dessert and cider apples has taken place (Morgan et al. 2002), the core collections we studied should be quite representative of the selection history of dessert and cider apples.

### SNP arrays

Genomic DNA was extracted from leaves of the 96 individuals using the NucleoSpin® Plant II kit (Macherey-Nagel GmbH and Co KG, Düren, Germany). Because apple leaves are full of polysaccharides and phenols that contaminate the extracted DNA and may prevent hybridization on the array, DNA samples were purified as follows: 0.1 volume of sodium acetate (final concentration 0.3 m), 2.5 volumes of cold 100% ethanol, and 1 μL of glycogen were added, the tubes were centrifuged at 13 000 *g* for 30 min, the supernatant was discarded, 200 μL of 70% ethanol was added, the tubes were centrifuged at 13 000 *g* for 10 min, the supernatant was discarded, the tubes were air-dried overnight, and the DNA was resuspended in the appropriate volume of water. DNA samples were then checked for quality using Nanodrop 1000 (Thermo Scientific, Wilmington, DE, USA), quantified using PicoGreen® (Invitrogen, Grand Island, NY, USA), and processed onto the International RosBREED SNP Consortium (IRSC) apple 8k SNP array v1 (Chagne et al. 2012a) following the Illumina® protocol.

### SNP filtering

SNPs were filtered using the Genotyping Module (version 1.8.4) of the Illumina® GenomeStudio software (Illumina Inc., San Diego, CA, USA). A visual inspection of each SNP was performed, and SNPs exhibiting a good genotypic clustering in distinct spots were kept. Paralogous SNPs were removed by performing BLAST onto the apple genome and removing probes having two equally good best hits onto the reference genome. This step was necessary for avoiding potential paralogy, due to the whole-genome duplication having occurred in the apple evolutionary history (Velasco et al. 2010). There were a few missing data in the dataset obtained from GenomeStudio, we therefore used fastPHASE 1.2 (Scheet and Stephens 2006) with the default parameters, and we indicated whether an individual belonged to the cider subgroup or to the dessert one to impute the missing data and to phase the SNPs belonging to a given linkage group (LG). Because the core collections were designed to maximize the genetic diversity and because SNPs for the 8k array were chosen among the most polymorphic markers in 27 dessert apple genomes (Chagne et al. 2012a), the allelic frequencies obtained may be biased compared to the full genetic pools of dessert and cider apples. Therefore, we excluded analyses based on the site frequency spectrum and focused only on analyses less sensitive to such biases.

### Estimation of linkage disequilibrium

The levels of linkage disequilibrium were estimated using the *r*^2^ parameter between all pairwise comparisons using the Haploview 4.2 software (Barrett et al. 2005) and a minor allele frequency (MAF) cutoff of 0.01. A first analysis was run without taking into account the structure and kinship in the collections; the levels of linkage disequilibrium were then corrected for population structure (see below) and kinship using the R package LDcorSV (Mangin et al. 2012). The kinship matrix, reflecting the degree of genetic covariance among individuals, was calculated with the Cocoa 1.1 software (Maenhout et al. 2009).

### Analysis of population structure

The ADMIXTURE 1.23 software (Alexander et al. 2009) was used to investigate the genetic population structure in the dataset. The number of genetic clusters *K* was assessed using values ranging from 1 to 10, and we chose the number of clusters for which the cross-validation error was the lowest. The cross-validation procedure masks one-fifth of the genotypes (five runs altogether) and calculates estimates for these genotypes. Each genotype is then predicted, and the software calculates a prediction error across all masked genotypes. The *Q* matrix, that is, the posterior probabilities for each individual to belong to a given cluster, outputted by ADMIXTURE 1.23 was used for the genotype–phenotype association analysis.

### Differentiation between cider and dessert apples – Detection of outlier loci

Pairwise single locus *F*_ST_ between the two core collections was calculated using either GENETIX 4.05 (Belkhir et al. 1996) or BayeScan 2.1 (Foll and Gaggiotti 2008). The Bayesian method implemented in the latter (Beaumont and Balding 2004) was run to detect outlier loci using the following parameters: after 20 pilot runs of 50 000 iterations and an additional burn-in of 500 000 iterations, we used 3 000 000 iterations (thinning interval of 50 and sample size of 50 000).

### Phenotype–genotype association

A genome-wide association study (GWAS) was run using the univariate linear mixed model (LMM) implemented in GEMMA (Zhou and Stephens 2012), taking into account the centered kinship matrix (*K*) calculated in GEMMA and the *Q* matrix from ADMIXTURE. A first analysis was performed on the two core collections by giving cider cultivars a score of 1 and dessert cultivars a score of 0. However, because not all cider cultivars are bitter, a second analysis was performed, this time not considering the cider versus dessert cultivars classification, but instead the bitterness of the cider apple cultivars, as recorded in the literature (Boré and Fleckinger 1997): bitter cider cultivars were given a score of 1 while sweet cider cultivars and dessert cultivars were given a score of 0. Both binary situations were treated as quantitative traits, as the linear mixed model is recognized as a robust approximation of a generalized linear model (Zhou et al. 2013). Markers were considered significantly associated with the phenotype for *P*-value ≤ 10^−3^. *P*-values obtained from GEMMA were used in R environment using the qqman package to generate a Manhattan plot (Turner 2014).

### Identification of candidate genes

The online apple genome browser hosted on http://www.rosaceae.org/, containing the gene model predictions made on the apple genome sequence, was used to investigate the putative functions of genes present in the genomic regions detected in the tests above. The Blast2Go 3.0 software (Conesa et al. 2005) was used to perform BLASTX on these sequences with a maximum Blast ExpectValue of 10^−3^. After gene ontology (GO) functional annotation, the KEGG tools were used to visualize the corresponding metabolic pathways. A BLASTN was run, and its results were used as inputs in Blast2GO 3.0 to retrieve GO annotations for the entire gene set of the apple genome. The regions of interest were then tested for enrichment of particular gene functions.

## Results

### SNP genotyping

**Figure 1:**
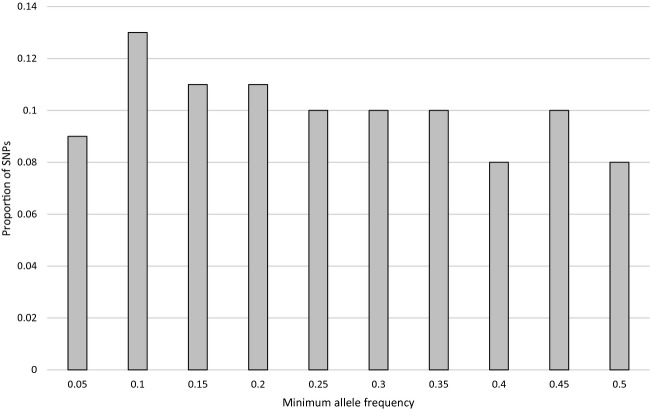
Allele frequency spectrum of the 3704 SNP markers of the array.

After visually screening the 7867 SNPs of the IRSC apple 8K SNP array v1 on GenomeStudio, a set of 4234 polymorphic SNPs evenly spread across the apple genome was obtained; after removing potential paralogous SNPs, the number of markers was reduced down to 3704. The number of markers per linkage group was approximately proportional to their length. The average distance between two adjacent SNPs was 140 kb, with the maximum distance separating markers ranging from 1.26 Mb on LG17 to 4.25 Mb on LG15. The distribution of the SNP minor allele frequencies (MAF) was quite uniform across the different possible MAF values (Fig.1) whether considering the cider or the dessert cultivars. Overall, few data were missing in the dataset, with 2981 markers having no missing data at all and the maximum percentage of missing data being 5.2% and 10.2% per marker and per individual, respectively. This made the inferences using fastPHASE 1.2 highly reliable.

### Estimation of linkage disequilibrium

**Figure 2:**
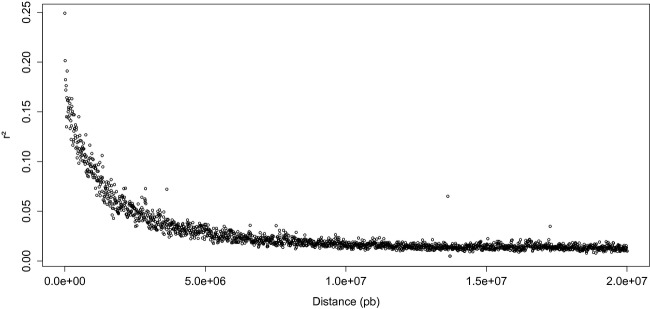
Decay of average linkage disequilibrium (measured as *r*^2^) versus physical distance in increments of 10 000 bp. Both core collections, cider and dessert, were included in the analysis because no difference was observed when correcting for structure and/or kinship.

The nonlinear regression model used to analyze the decay of linkage disequilibrium (LD) with the physical distance showed that the squared allele correlation parameter *r*^2^ decayed below 0.2 within 100 kb (Fig.2). When analyzed separately, the cider and the dessert core collections showed very similar behaviors. The results obtained on the whole dataset when taking into account kinship or/and population structure were very similar too. We therefore assumed that loci distant from more than 100 kb were not in LD and considered windows of 100 kb on both sides of outlier SNPs for finding candidate genes possibly evolving under divergent selection.

### Differentiation between cider and dessert apples – Analysis of population structure

**Figure 3:**
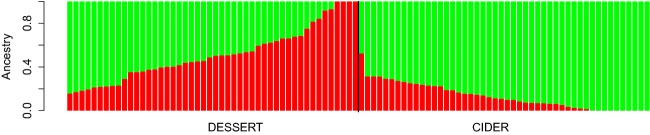
Population structure of 96 apple cultivars from the cider and dessert genetic pools. Membership probabilities were obtained with ADMIXTURE for *K* = 2. The bar plot, generated using the qqman package in R, shows each individual as a vertical bar.

Pairwise *F*_ST_ between cider and dessert apples ranged from 0 to 0.24, with a mean value of 0.014, confirming the weak differentiation between the two core collections. ADMIXTURE analyses revealed a minimum value of the cross-validation error for *K* = 2. Only a quarter of the individuals actually showed a clear assignment (membership probability >0.9) to any cluster, supporting the lack of further structure in the dataset. The *Q* matrix for *K* = 2 (Fig.3) confirms the lack of strong differentiation according to the cider/dessert classification, even using genome-wide markers.

### Detection of *F*_ST_ outlier loci and genotype–phenotype associations

**Figure 4:**
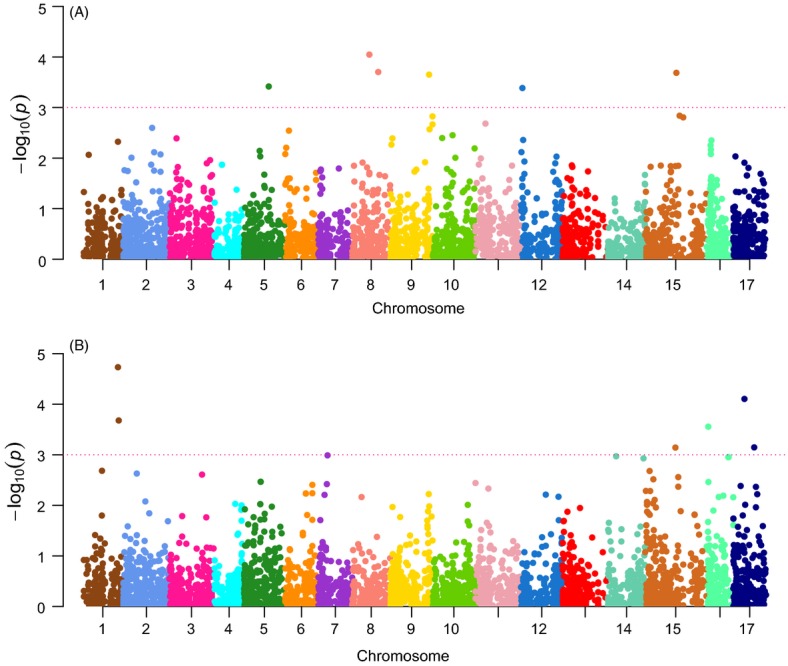
Manhattan plot of the GWAS testing for association between genotypes and the cider/dessert (A) or bitter/sweet phenotype (B). The –log10 of the *P*-value of 3704 SNPs after correction for structure and kinship is plotted against the physical position. SNPs above the blue line are those exhibiting significant *P*-values and thus associated with the cider/dessert or bitter/sweet phenotype.

Of the 3704 SNPs tested for their probability to have been under divergent selection using Bayescan 2.1, five exhibited significant genetic differentiation. These five outlier SNPs were located as follows: one SNP on LG08 at 11.32 Mb, two SNPs on LG15 at 26.38 Mb and 29.20 Mb, and two SNPs on LG17 at 10.30 Mb (Table1). The GWAS testing SNP association with cider/dessert variety types revealed six SNPs with significant *P*-values (i.e., −Log10 *P*-value ≥ 3). These markers were located as follows: one SNP on LG05 at 19.23 Mb, two SNPs on LG08 at 13.05 Mb and 19.85 Mb, one SNP on LG09 at 29.70 Mb, one SNP on LG12 at 1.03 Mb, and one SNP on LG15 at 23.86 Mb (Table2 and Fig.4A). The bitter/sweet trait was found significantly associated with six SNPs located as follows: 2 SNPs on LG01, respectively located at 2.61 Mb and 2.67 Mb, one SNP on LG15 at 23.12 Mb, one SNP on LG16 at 1.45 Mb, and two SNPs on LG17, respectively located at 8.42 Mb and 15.88 Mb (Table2 and Fig.4B).

**Table 1:** SNPs showing significant levels of *F*_ST_ detected by BayeScan 2.1

SNP Name	LG	Position	*F*_ST_
GDsnp01132	8	11 328 418	0.23
RosBREEDSNP_SNP_CA_29926704_Lg15_RosCOS1232_MAF50_MDP0000283141_exon1	15	26 329 550	0.23
RosBREEDSNP_SNP_AG_33667246_Lg15_01897_MAF40_151341_exon1	15	29 068 827	0.24
RosBREEDSNP_SNP_CT_10901071_Lg17_00918_MAF10_1668766_exon3	17	10 334 128	0.19
RosBREEDSNP_SNP_CT_10898449_Lg17_00918_MAF10_466062_exon6	17	10 336 750	0.19

LG, Linkage Group.

**Table 2:** SNPs showing significant association with the cider/dessert or bitter/sweet phenotypes when taking into account structure and kinship between individuals using GEMMA

SNP Name	LG	Position	*P*-value
RosBREEDSNP_SNP_CT_22024068_Lg5_RosCOS3072_MAF30_MDP0000753788_exon2[Table-fn tf2-2]	5	19 238 624	3.83 × 10^−4^
RosBREEDSNP_SNP_TC_15251985_Lg8_00354_MAF10_753213_exon1[Table-fn tf2-2]	8	13 053 086	8.98 × 10^−5^
RosBREEDSNP_SNP_TG_23835076_Lg8_RosCOS3331_MAF40_488673_exon1[Table-fn tf2-2]	8	19 848 379	1.99 × 10^−4^
RosBREEDSNP_SNP_GA_33077622_Lg9_01200_MAF20_MDP0000613052_exon1[Table-fn tf2-2]	9	29 701 351	2.24 × 10^−4^
RosBREEDSNP_SNP_GA_1240623_Lg12_RosCOS3293_MAF40_1686868_exon1[Table-fn tf2-2]	12	1 033 191	4.12 × 10^−4^
RosBREEDSNP_SNP_AG_27056933_Lg15_02084_MAF30_1677692_exon1[Table-fn tf2-2]	15	23 859 694	2.06 × 10^−4^
RosBREEDSNP_SNP_AG_32748739_Lg1_RosCOS2753_MAF10_520680_exon1[Table-fn tf2-3]	1	26 153 648	1.86 × 10^−5^
RosBREEDSNP_SNP_AC_33325153_Lg1_01951_MAF10_132337_exon1[Table-fn tf2-3]	1	26 730 062	2.10 × 10^−4^
GDsnp01850[Table-fn tf2-3]	15	23 124 410	7.20 × 10^−4^
RosBREEDSNP_SNP_AC_1452699_Lg16_MDP0000303483_MAF50_MDP0000303483_exon2[Table-fn tf2-3]	16	1 452 699	2.78 × 10^−4^
RosBREEDSNP_SNP_CT_8827345_Lg17_01842_MAF30_MDP0000891106_exon4[Table-fn tf2-3]	17	8 427 545	7.87 × 10^−5^
RosBREEDSNP_SNP_CT_17294445_Lg17_01964_MAF10_1662340_exon9[Table-fn tf2-3]	17	1 588 1764	7.14 × 10^−4^

LG, Linkage Group.

SNP associated with the cider/dessert phenotype.

SNP associated with the bitter/sweet phenotype.

### Genes around candidate SNPs associated with phenotypes

We looked at the gene predictions available on the first version of the genome of apple within 200 kb around the seventeen SNPs detected above as putatively under diversifying selection. In the regions containing the five *F*_ST_ outlier loci detected by BayeScan, 85 predicted genes were found, whose main classes of putative functions are reported in Supporting information. In the 12 regions carrying the markers found to be associated with the cider/dessert or bitter/sweet phenotypes, 179 and 156 predicted genes were found respectively, whose main classes of putative functions are shown in Supporting information. Among these genes, the most represented biological processes were as follows: (i) amino acid metabolism and starch and sugar metabolism for the *F*_ST_ outliers, (ii) nucleotide metabolism and glycerolipid metabolism for the variety type associated regions, and (iii) purine metabolism and thiamine metabolism for the bitterness associated regions. The enrichment test made using the entire predicted gene set as reference did not yield any significant result.

## Discussion

### Possible biases due to sample and marker choices

We used in this study core collections that maximize the genetic diversity present in larger initial collections, which may have generated biases in allelic frequencies. However, *F*_ST_ outliers and GWAS methods should be robust to such biases, and even conservative. Indeed, core collections balance the initial extreme allelic frequencies, so that association should be valid across even more diverse genotypes to be significant in core collections. The use of core collections instead of random sampling may in addition have led to an underestimation of linkage disequilibrium. Indeed, the increased distances between accessions within a core collection reflect an increased number of generations from the most recent common ancestor and thus a higher number of crossing-overs between linked loci (Nordborg and Tavare 2002). The LD values in the core collection are, however, again conservative and are actually the appropriate estimates to consider for the definition of the window size around the significant SNPs in the core collections.

Possible ascertainment biases in the SNP array design result from the choice of the markers among the most polymorphic SNPs based on genome resequencing of 27 dessert apple cultivars (Chagne et al. 2012a). The direct consequence is a more uniform distribution of the MAF spectrum than generally observed for resequencing data (Pe’er et al. 2006). This SNP ascertainment bias most probably led to overestimating the *r*^2^ values (Nielsen and Signorovitch 2003; Nielsen 2004; Lachance and Tishkoff 2013) and thus the LD extent. In the end, the combined impact of the core collection sampling and the SNP ascertainment bias on the LD estimation is difficult to assess. In addition, SNPs exhibiting contrasted frequency in the dessert and cider apple pools may have been discarded from the 8k apple array, even if they had a higher frequency in the cider apple gene pool, thus restricting the chance of detecting the corresponding genomic regions. These ascertainment biases, however, are again conservative: they may have led us to miss some genomic regions involved in cider versus dessert cultivars, but should not have yielded false positives. The regions detected here should therefore be considered as interesting candidates, but not an exhaustive list.

### Low level of genomic differentiation between cider and dessert variety types

A previous study had reported a lack of population genetic structure between cider and dessert apples, using only a couple of dozen of microsatellite markers (Cornille et al. 2012). Our results confirm this result using a much higher number of markers of a different type (i.e., SNPs instead of SSR) along the genome, with no clear assignment of most of the different cultivars to either one or the other of the two inferred clusters according to their variety type. The low mean value of *F*_ST_ between cider and dessert apples (0.014 in our study) also supports the lack of genome-wide differentiation and is consistent with the mean *F*_ST_ value of 0.02 found by Cornille et al. (2012). Actually, some cultivars, discarded from the present study, are known to be used for both cider and dessert (e.g., Bagué Petit, Raccroupi, Cazo Jaune), which means the phenotypic classification in cider and dessert apples is not morphologically clear-cut either.

### Long distance LD in the cultivated apple

The *r*^2^ was found here to decrease below 0.2 within 100 kb. In previous studies on apples, *r*^2^ was found decaying below 0.2 within 500 kb in a population of 7 full-sib families genotyped with 2500 SNPs (Kumar et al. 2012) and within 1 cM (corresponding to approximately 500 kb considering that the apple genome is 750 Mb and that the genetic map is 1500 cM long) in a collection of 132 apple cultivars genotyped with 238 SNPs (Micheletti et al. 2008). Such discrepancies with our study may be explained by a sampling of siblings in the former study therefore implying fewer recombination events than in a core collection encompassing a high diversity and several generations between individuals. In the latter study, a fewer number of SNPs, not spanning the entire genome, is also an explanation for a larger range of LD.

Linkage disequilibrium has also been studied in other *Rosaceae* crops such as *Prunus persica*, where *r*^2^ reached 0.1 within 1200 kb in an Oriental peach germplasm (Li et al. 2013), and *Pyrus pyrifolia*, where *r*^2^ fell below 0.2 at approximately 1800 kb in a population of old and modern cultivars, considering the pear genome is 600 Mb and 1100 cM long (Iwata et al. 2013). Studies performed on other allogamous tree species showed lower values of distances above which the LD decayed below 0.2: 200 bp in *Populus tremula* (Ingvarsson 2005) and approximately 2 kb in *Pinus taeda* L. (Brown et al. 2004). These levels of LD appear low compared to our results, probably because the studies were conducted on wild populations of forest trees, in which a much higher number of recombination events probably occurred since the last population bottleneck. In addition, the rather high average distance between our markers may have led to miss some occurrences of short-distance LD.

### Differentiated genomic regions between cider and dessert apples

We identified here a total of 17 regions potentially bearing genes responsible for phenotypic differences between cider and dessert apples. Five of these regions harbored *F*_ST_ outlier loci that exhibited high differentiation levels between cider and dessert cultivars while the other twelve showed significant associations between the genotypic information and the variety type or the bitter trait while accounting for structure and kinship. According to the results on LD decay, 200 kb windows around the significant SNPs were investigated. The enrichment test performed on the three set of genes around significant SNPs, that is, *F*_ST_ outliers and the two association analyses results, did not detect any particularly overrepresented pathway. No genes known to be involved in the traits differentiating cider and dessert cultivars, such as the polyphenol pathway, were identified around the *F*_ST_ outliers located on LG08 and LG15. Two genes having high sequence similarity with UDP-glycosyltransferases were found around the two outlier markers on LG17. These genes can play a role in the synthesis pathways of several polyphenol compounds such as flavonoids or anthocyanidins, as exemplified by the *MdPT1* gene (Jugdé et al. 2008) involved in the glycosylation of phloretin into phlorizin, a major dihydrochalcone of apple known to have a bitter taste that may contribute to the peculiar flavor of cider (Whiting and Coggins 1975).

Regarding the results of the association between the genotypic information and the variety type, the two SNPs located on LG08 colocalized with QTLs linked to biennial bearing and yield (Guitton et al. 2012). Cider apples are in fact known to be more subject to biennial bearing than dessert apples (Dapena et al. 2005). However, no gene known to control any traits *a priori* differentiating cider and dessert apples was found within the genomic regions examined around the six SNPs detected as significantly associated with the variety type. This may be due to lack of knowledge on these genes, and actually 13% of the genes did not have any predicted function. Alternatively, this may be because selection targeted the regulatory elements in the pathways. In fact, several genes coding for transcriptional regulation elements were found in these candidate regions. Finally, the estimation we made on the extent of LD may not reflect reality in these particular regions (as it is a genome-wide mean value we calculated) and could lead the causative factors for our outliers to be located outside of the windows examined.

All the six genomic regions identified when testing the association between the genotypes and the bitter/sweet phenotype were found to colocalize with QTLs responsible for the content of several polyphenolic compounds, either measured in the flesh or measured in the peel of the fruits (Chagne et al. 2012b; Khan et al. 2012b; Kumar et al. 2012; Verdu et al. 2014). The two SNPs located on LG01 colocalized with three QTLs responsible for *p*-coumaroyl quinic acid, hydroxycinnamic acid, and flavonols contents. The SNP located on LG15 colocalized with two QTLs responsible for flavonols and flavonols contents and the two SNPs on LG17, respectively, colocalized with QTLs responsible for quercetin 3-*O*-rutinoside and chlorogenic acid contents. The last area located on LG16 colocalized with a region well known to host several strong effect QTLs responsible for numerous polyphenolic compounds such as catechin, epicatechin, and procyanidins, all belonging to the flavonol class of polyphenols (Chagne et al. 2012b; Khan et al. 2012b). A gene coding for a *LeucoAnthocyanidin Reductase* (*LAR*) was identified underlying this QTL hotspot and is thought to be the gene responsible for the numerous QTLs in this area (Khan et al. 2012a). The *LAR* gene is indeed the one in the polyphenol pathway leading to the formation of the flavonols from leucocyanidin. Interestingly, the SNP significantly associated with the bitter/sweet phenotype and located on LG16 at 1.43 Mb was close to the *LAR* gene (MDP0000376284) located at 1.53 Mb, which makes our result highly consistent with this particular QTL hotspot and the *LAR* candidate gene. Altogether, the six SNPs associated to the bitter/sweet phenotype were located very close (less than 1 Mb on average) to the markers exhibiting the highest LOD score in the QTL analyses.

### Applications in cider apple breeding

This study is a first step for the identification of the genetic bases of phenotypic traits that differentiate cider and dessert apple varieties. In addition, our markers will be useful for marker-assisted selection (MAS) for breeding cider varieties carrying both traits already present in cider varieties (such as high polyphenol content) and traits mainly present in dessert apple varieties (such as annual bearing, high yield, or disease resistance). Our markers can indeed guide both the choice of the cider apple progenitors and the selection of seedlings from crosses between dessert and cider varieties and thus segregating for the favorable haplotypes. By genotyping the seedlings of a cross between a cider and a dessert variety type at the loci we identified as linked with traits of interest, one could choose the individuals bearing the favorable alleles and keep the individuals combining traits from the two variety types. Another application of the information we described here could be the inventory of the several traits and genomic regions responsible for them to better manage germplasm diversity in the cultivated apple (Prada 2009).

## Conclusions

Unraveling the genomic bases of quantitative trait variation is essential for understanding evolution and for accelerating plant breeding (Alonso-Blanco and Méndez-Vigo 2014). Furthermore, the question of sustainable management of germplasm resources is increasingly recognized as a fundamental goal to achieve in many crops (see the DivSeek initiative, http://www.divseek.org/). Recently, it has been suggested that an international consortium for the sustainable management of apple genetic diversity in particular is timely (Volk et al. 2014). Our results on the detection of a few key genomic regions involved in the phenotypic differentiation between cider and dessert apples emerging from an otherwise homogeneous genomic background should be very useful for designing such sustainable apple program. The identified outlier genomic regions will indeed be good targets for screening important genetic variation for conserving both cider and dessert apples specific traits. These programs should also focus on the sustainable conservation of the wild apple gene pools. Wild-to-crop introgressions have indeed been a key driver of the cultivated apple evolution, particularly through introgression from the European crabapple *M. sylvestris* (Cornille et al. 2012). It would be interesting to assess whether the outliers detected here in the cultivated apple have originated from such introgressions from the bitter crabapples. It would feature wild gene pools as sources of key genes for cultivated apple breeding in cider and dessert apples. Overall, our results thus illustrate how genomic can help to feed breeding and conservation programs, and a similar approach could be developed for detecting the genomic basis of other key traits, such as resistance to pathogens or climate adaptation.

## Data archiving statement

The genotypic data have been deposited on http://www.rosaceae.org/search/diversity: Accession Number tfGDR1016.

## Supporting Information

**Table S1.** Names of the 96 cultivars chosen from the INRA Angers collections of old cider and dessert apple varieties.

**Table S2.** Results from the Blast2GO software on the genes identified around the significant SNPs.
