# Supplementary material for: Genomic basis of the differences between cider and dessert apple varieties
Source: Evol Appl. 2015 Jun 13;8(7):650–61. doi: 10.1111/eva.12270 (PMC4516418; doi:10.1111/eva.12270)
Supplement: Supplementary file 1 [file eva0008-0650-sd1.docx]

**Table 1.** Names of the 96 cultivars chosen from the INRA Angers collections of old cider and dessert apple varieties

| Name of the variety | Type |
| --- | --- |
| AMADOU | Dessert |
| API | Dessert |
| BARBE | Dessert |
| BEACON | Dessert |
| BEAUTY OF BATH | Dessert |
| BELLE DE MAGNI | Dessert |
| BELLE FILLE DE L'INDRE | Dessert |
| BELLE FILLE DE ST FEYRE | Dessert |
| BELLE FLEUR KRASNYI | Dessert |
| BORDES | Dessert |
| BOROWITSKY | Dessert |
| CACHAO SAGARRA | Dessert |
| CALVILLE DE MLEIEV | Dessert |
| CALVILLE DU ROI | Dessert |
| COLATE | Dessert |
| DE BONDE | Dessert |
| DE NOEL | Dessert |
| DIRECTEUR LESAGE | Dessert |
| ELLISON'S ORANGE | Dessert |
| ENTZEA SAGARRA | Dessert |
| FEVRETTE | Dessert |
| FLEURITARD ROUGE | Dessert |
| GELADE | Dessert |
| GEWURTZLUIKEN | Dessert |
| GIREUSE | Dessert |
| GRAIN D'OR DES CHARENTES | Dessert |
| GRENADIER | Dessert |
| GROSSE SAINT CLEMENT | Dessert |
| MERVEILLE DE VITRY | Dessert |
| PETIT API | Dessert |
| PETIT MUSEAU DE LIEVRE | Dessert |
| PETITE MADELEINE | Dessert |
| PIGEON DE JERUSALEM | Dessert |
| POMME BLANCHE D'ETE | Dessert |
| POMME D'ETE ROUGEUR DE PECHE | Dessert |
| POMME FER | Dessert |
| POMME ORANGE | Dessert |
| POMME ST JACQUES | Dessert |
| REINETTE DE PLUVIGNE | Dessert |
| REINETTE ETOILEE | Dessert |
| REINETTE SANGUINE DU RHIN | Dessert |
| ROMARIN | Dessert |
| ROUMENTINE | Dessert |
| SAINT MICHEL | Dessert |
| TAFFETAS BLANC | Dessert |
| TETON DE DEMOISELLE | Dessert |
| TRANSPARENTE BLANCHE | Dessert |
| TRELAGE | Dessert |
| ABONDANCE | Cider |
| AMERE NOUVELLE | Cider |
| AMERE ST JACQUES | Cider |
| AVROLLES GROSSE | Cider |
| BINET BLANC | Cider |
| BLANC JAUNET | Cider |
| BLANC MOLLET | Cider |
| BLANCHET | Cider |
| CARISI | Cider |
| CHUERO RU | Cider |
| CROLLON | Cider |
| CUL D'OISAN | Cider |
| DAMELOT | Cider |
| DOUCE COET LIGNE | Cider |
| DOUCE ROUSSE | Cider |
| DOUX CORIER | Cider |
| DOUX LOZON | Cider |
| Dx V CARROUGES | Cider |
| GALOPIN | Cider |
| GENERAL | Cider |
| GRISE DIEPPOIS | Cider |
| GUILLEVIC | Cider |
| HERBAGE SEC | Cider |
| MANERBE | Cider |
| MARECHAL | Cider |
| MAUGERE | Cider |
| MERISIER | Cider |
| MONTE EN HAUT | Cider |
| MOULIN A VENT EURE | Cider |
| MUSCADET DE DIEPPE | Cider |
| NOE BINAY | Cider |
| P.G.R. JANZE | Cider |
| PERICO | Cider |
| PETIT DOUX DE BRETAGNE | Cider |
| PETIT FREQUIN ROUGE | Cider |
| PETIT JAUNE | Cider |
| PETIT MARIN ONFROY | Cider |
| PETITE SORTE | Cider |
| PIERRE ALLAIRE | Cider |
| POMME DE MOET | Cider |
| PORTIER | Cider |
| RADOR | Cider |
| REINETTE MARBREE DE LUZOIR | Cider |
| RENAO | Cider |
| RENAO PETIT | Cider |
| RENE MARTIN | Cider |
| ROUGET DE DOL | Cider |
| SAINT BAZYL | Cider |
