# Supplementary material for: Genomic basis of the differences between cider and dessert apple varieties
Source: Evol Appl. 2015 Jun 13;8(7):650–61. doi: 10.1111/eva.12270 (PMC4516418; doi:10.1111/eva.12270)
Supplement: Supplementary file 2 [file eva0008-0650-sd2.docx]

**Table 2.** Results from the Blast2GO software on the genes identified around the significant SNPs.

| Trait | Seq. Name | Seq. Description | #Hits | min. eValue | MS^a^ | GOs^b^ | Enzyme Codes |
| --- | --- | --- | --- | --- | --- | --- | --- |
| FST | MDP0000076511 | endoplasmic reticulum-golgi intermediate compartment protein 3-like | 2.00E+01 | 0.00% | 91.30% | C:endoplasmic reticulum | - |
|  | MDP0000084203 | vacuolar-processing enzyme-like | 20 | 0.00% | 94.35% | P:biological_process; F:peptidase activity | EC:3.4.22; EC:3.4 |
|  | MDP0000126636 | probable protein phosphatase 2c 72 | 20 | 0.00% | 80.20% | P:biological_process; F:molecular_function | - |
|  | MDP0000131883 | ---NA--- | 0 |  |  | - |  |
|  | MDP0000133911 | udp-glycosyltransferase 85a2-like | 20 | 0.00% | 84.85% | F:transferase activity, transferring hexosyl groups; P:metabolic process | - |
|  | MDP0000136026 | nedd8 ultimate buster 1 | 2.00E+01 | 1.18E-180 | 92.10% | F:protein binding | - |
|  | MDP0000139577 | PREDICTED: uncharacterized protein LOC103426009 | 20 | 5.75E-162 | 77.05% | - |  |
|  | MDP0000149647 | nodulation receptor kinase-like | 2.00E+01 | 6.48E-92 | 94.25% | P:signal transduction; F:ion binding; P:cellular protein modification process; P:cellular amino acid metabolic process; C:cellular_component; F:signal transducer activity; F:kinase activity | EC:2.7.11.25; EC:2.7.11 |
|  | MDP0000150721 | udp-glycosyltransferase 85a2-like | 20 | 0.00% | 85.10% | P:metabolic process; F:transferase activity, transferring hexosyl groups | - |
|  | MDP0000150726 | e3 ubiquitin-protein ligase march10 | 2.00E+01 | 0.00% | 75.15% | F:ion binding | - |
|  | MDP0000150727 | vacuolar protein sorting-associated protein 53 homolog | 20 | 0.00% | 93.60% | - |  |
|  | MDP0000150729 | chlorophyll a-b binding protein chloroplastic | 2.00E+01 | 0.00% | 93.00% | P:generation of precursor metabolites and energy; P:cellular protein modification process; P:photosynthesis; C:protein complex; C:cellular_component; C:plastid; F:molecular_function; C:thylakoid | - |
|  | MDP0000159850 | clathrin interactor epsin 1-like | 20 | 0.00% | 84.85% | C:intracellular; F:molecular_function | - |
|  | MDP0000168905 | nodulation receptor kinase-like | 2.00E+01 | 3.28E-55 | 56.65% | F:molecular_function | EC:2.7.11 |
|  | MDP0000172806 | gibberellin 3-beta-dioxygenase 1-like | 17 | 8.32E-08 | 69.94% | P:oxidation-reduction process; F:oxidoreductase activity, acting on paired donors, with incorporation or reduction of molecular oxygen, 2-oxoglutarate as one donor, and incorporation of one atom each of oxygen into both donors; F:oxidoreductase activity; F:gibberellin 3-beta-dioxygenase activity; F:iron ion binding; P:response to gibberellin; P:response to red light; P:response to red or far red light; F:transcription factor binding | |
|  | MDP0000173095 | mtd1 family protein | 2.00E+01 | 6.72E-144 | 73.35% | - |  |
|  | MDP0000175487 | cmp-sialic acid transporter 5-like | 20 | 3.18E-143 | 93.20% | P:transmembrane transport; F:transmembrane transporter activity; C:cytoplasm; P:anatomical structure formation involved in morphogenesis; C:Golgi apparatus; C:cellular_component; P:transport | - |
|  | MDP0000179065 | oxidation resistance protein 1-like | 20 | 0.00% | 81.10% | - |  |
|  | MDP0000181379 | tubulin-folding cofactor c-like | 2.00E+01 | 5.99E-19 | 70.05% | P:biological_process | - |
|  | MDP0000191944 | calcium uptake protein mitochondrial-like | 2.00E+01 | 4.32E-41 | 54.60% | F:calcium ion binding | - |
|  | MDP0000191948 | protein unc-45-a-like protein | 2.00E+01 | 4.84E-85 | 91.80% | F:protein binding | - |
|  | MDP0000192297 | myb family transcription factor-related protein | 2.00E+01 | 1.89E-107 | 58.85% | C:nucleus; F:DNA binding; P:sulfur compound metabolic process; F:nucleic acid binding transcription factor activity; P:anatomical structure development; P:cellular amino acid metabolic process; P:cellular nitrogen compound metabolic process; P:biosynthetic process; F:molecular_function; P:response to stress | - |
|  | MDP0000197740 | f-box kelch-repeat protein at3g06240-like | 20 | 1.07E-37 | 46.45% | - |  |
|  | MDP0000200785 | ras-related protein rabh1e | 2.00E+01 | 9.63E-149 | 98.15% | P:biological_process; P:signal transduction; F:ion binding; P:transport; F:molecular_function | EC:3.6.1; EC:3.6.1.15 |
|  | MDP0000201472 | l-lactate dehydrogenase a | 20 | 1.77E-12 | 80.65% | P:small molecule metabolic process; P:carbohydrate metabolic process; P:biological_process; P:generation of precursor metabolites and energy; C:cytoplasm; F:oxidoreductase activity; P:catabolic process | EC:1.1.1.27 |
|  | MDP0000201500 | transmembrane 9 superfamily member 4 | 20 | 0.00% | 91.55% | C:endosome; C:cytoplasm; C:Golgi apparatus; C:vacuole; C:cellular_component | - |
|  | MDP0000208497 | plant t5j17-70 | 2.00E+01 | 2.41E-108 | 75.95% | - |  |
|  | MDP0000213515 | btb poz domain-containing protein dot3 | 2.00E+01 | 0.00% | 83.75% | P:biological_process; P:anatomical structure development | - |
|  | MDP0000221796 | cmp-sialic acid transporter 5-like | 2.00E+01 | 4.47E-140 | 93.65% | P:transmembrane transport; F:transmembrane transporter activity; C:cytoplasm; P:anatomical structure formation involved in morphogenesis; C:Golgi apparatus; C:cellular_component; P:transport | - |
|  | MDP0000222448 | ---NA--- | 0 |  |  | - |  |
|  | MDP0000222909 | asparagine synthetase | 20 | 4.25E-76 | 92.60% | F:ion binding; P:cellular amino acid metabolic process; P:cellular nitrogen compound metabolic process; P:biosynthetic process; F:ligase activity | EC:6.3.5.4 |
|  | MDP0000225641 | glycosyl hydrolase family 10 protein carbohydrate-binding domain-containing protein isoform 1 | 20 | 0.00% | 87.15% | P:carbohydrate metabolic process; P:catabolic process; P:cell wall organization or biogenesis; F:hydrolase activity, acting on glycosyl bonds | - |
|  | MDP0000232773 | portal 56 | 20 | 0.00% | 79.25% | - |  |
|  | MDP0000232774 | flowering time control protein fpa | 20 | 0.00% | 52.10% | F:nucleic acid binding; F:nucleotide binding | |
|  | MDP0000235629 | nodulation receptor kinase-like | 20 | 0.00% | 71.00% | P:biological_process; P:cellular amino acid metabolic process; C:cellular_component; F:kinase activity; F:molecular_function | EC:2.7.11 |
|  | MDP0000240075 | nodulation receptor kinase-like | 2.00E+01 | 1.60E-73 | 94.30% | P:signal transduction; F:ion binding; P:cellular protein modification process; P:cellular amino acid metabolic process; C:cellular_component; F:signal transducer activity; F:kinase activity | EC:2.7.11.25; EC:2.7.11 |
|  | MDP0000241162 | vacuolar-processing enzyme-like | 20 | 0.00% | 86.75% | P:biological_process; F:peptidase activity | EC:3.4.22; EC:3.4 |
|  | MDP0000241165 | PREDICTED: uncharacterized protein LOC103440997 | 20 | 2.45E-180 | 88.25% | C:nucleus; C:protein complex; P:DNA metabolic process; P:response to stress | - |
|  | MDP0000241166 | serine threonine-protein kinase at5g01020 | 2.00E+01 | 1.35E-96 | 82.60% | F:ion binding; P:cellular protein modification process; P:cellular amino acid metabolic process; F:kinase activity | EC:2.7.10; EC:2.7.11; EC:2.7.10.2 |
|  | MDP0000243737 | cyclin-dependent kinase c-2-like | 20 | 0.00% | 77.75% | P:biological_process; F:kinase activity; F:molecular_function | EC:2.7.11 |
|  | MDP0000243738 | probable mitochondrial adenine nucleotide transporter btl3 | 20 | 0.00% | 83.95% | P:transmembrane transport; C:cellular_component; C:plastid | - |
|  | MDP0000244376 | ras-related protein rabh1e-like | 2.00E+01 | 1.71E-144 | 98.05% | P:biological_process; P:signal transduction; F:ion binding; P:transport; F:molecular_function | EC:3.6.1; EC:3.6.1.15 |
|  | MDP0000256619 | glucose-1-phosphate adenylyltransferase small chloroplastic amyloplastic | 2.00E+01 | 0.00% | 95.55% | P:carbohydrate metabolic process; F:nucleotidyltransferase activity; P:generation of precursor metabolites and energy; C:cytoplasm; P:anatomical structure development; C:protein complex; P:biosynthetic process; C:extracellular region; C:plastid | EC:2.7.7.27 |
|  | MDP0000258981 | nodulation receptor kinase-like | 2.00E+01 | 1.99E-70 | 69.90% | P:biological_process; F:kinase activity | - |
|  | MDP0000266156 | transcription factor rax2-like | 2.00E+01 | 2.64E-180 | 67.15% | F:chromatin binding; F:DNA binding; C:chromatin | - |
|  | MDP0000268708 | exonuclease 3 -5 domain-containing protein 1-like | 2.00E+01 | 1.73E-121 | 90.00% | F:RNA binding; F:nuclease activity; P:cellular nitrogen compound metabolic process | EC:3.1 |
|  | MDP0000275432 | glucan endo- -beta- basic isoform-like | 7 | 1.03E-52 | 66.00% | F:molecular_function | - |
|  | MDP0000280307 | homeobox protein knotted-1-like 2 | 20 | 0.00% | 87.80% | C:nucleus; F:DNA binding; F:nucleic acid binding transcription factor activity; P:cellular nitrogen compound metabolic process; P:biosynthetic process; C:protein complex; C:intracellular | - |
|  | MDP0000283141 | phosphoglucan phosphatase amyloplastic | 2.00E+01 | 7.17E-156 | 72.30% | P:carbohydrate metabolic process; P:biological_process; F:phosphatase activity; C:plastid | EC:3.1.3.16; EC:3.1; EC:3.1.3.41; EC:3.1.3 |
|  | MDP0000290409 | calmodulin-binding transcription activator 3 | 2.00E+01 | 0.00% | 80.00% | C:nucleus; F:DNA binding | - |
|  | MDP0000290414 | nedd8 ultimate buster 1 | 20 | 0.00% | 84.70% | F:protein binding | - |
|  | MDP0000290415 | protein os-9-like | 20 | 0.00% | 88.05% | P:catabolic process; C:endoplasmic reticulum; P:response to stress | - |
|  | MDP0000299712 | 03-mai | 20 | 0 | 81.00% | F:RNA binding; F:nuclease activity; P:cellular nitrogen compound metabolic process | EC:3.1 |
|  | MDP0000306669 | cmp-sialic acid transporter 5-like | 20 | 7.63E-178 | 94.05% | P:transmembrane transport; F:transmembrane transporter activity; C:cytoplasm; P:anatomical structure formation involved in morphogenesis; C:Golgi apparatus; C:cellular_component; P:transport | - |
|  | MDP0000306670 | asparagine synthetase | 2.00E+01 | 6.28E-80 | 86.60% | P:biological_process; P:sulfur compound metabolic process; F:ion binding; C:cytosol; P:cellular amino acid metabolic process; P:biosynthetic process; P:cellular nitrogen compound metabolic process; C:cellular_component; F:ligase activity; P:response to stress | EC:6.3.5.4; EC:6.3.1; EC:6.3.1.1 |
|  | MDP0000320591 | PREDICTED: uncharacterized protein LOC103952713 isoform X1 | 20 | 4.19E-27 | 71.20% | - |  |
|  | MDP0000324950 | ---NA--- | 0 |  |  | - |  |
|  | MDP0000332596 | serine mitochondrial | 20 | 0.00% | 95.20% | P:biological_process; F:ion binding; P:cellular amino acid metabolic process; F:methyltransferase activity; P:biosynthetic process; C:ribosome; C:extracellular region; C:plasma membrane; C:plastid; F:molecular_function; P:response to stress; C:thylakoid; C:nucleus; F:RNA binding; C:mitochondrion; P:cofactor metabolic process; C:cytosol | EC:2.1.2.1 |
|  | MDP0000332597 | serine mitochondrial | 20 | 0.00% | 95.20% | P:biological_process; F:ion binding; P:cellular amino acid metabolic process; F:methyltransferase activity; P:biosynthetic process; C:ribosome; C:extracellular region; C:plasma membrane; C:plastid; F:molecular_function; P:response to stress; C:thylakoid; C:nucleus; F:RNA binding; C:mitochondrion; P:cofactor metabolic process; C:cytosol | EC:2.1.2.1 |
|  | MDP0000336114 | ---NA--- | 0 |  |  | - |  |
|  | MDP0000340361 | ---NA--- | 0 |  |  | - |  |
|  | MDP0000369409 | ---NA--- | 0 |  |  | - |  |
|  | MDP0000392459 | ---NA--- | 0 |  |  | - |  |
|  | MDP0000404395 | transmembrane 9 superfamily member 4 | 20 | 0.00% | 91.35% | C:endosome; C:cytoplasm; C:Golgi apparatus; C:vacuole; C:cellular_component | - |
|  | MDP0000404396 | myb family transcription factor apl-like isoform x2 | 4.00E+00 | 1.58E-39 | 60.75% | F:DNA binding | |
|  | MDP0000500222 | f-box kelch-repeat protein at3g06240-like | 20 | 0.00% | 65.80% | F:protein binding | - |
|  | MDP0000503661 | ---NA--- | 0 |  |  | - |  |
|  | MDP0000529897 | asparagine synthetase | 20 | 7.60E-60 | 85.70% | F:ion binding; C:cytosol; P:cellular amino acid metabolic process; P:cellular nitrogen compound metabolic process; P:biosynthetic process; F:ligase activity | EC:6.3.5.4; EC:6.3.1; EC:6.3.1.1 |
|  | MDP0000543680 | formin-like protein 14 | 2.00E+01 | 2.61E-80 | 75.70% | - |  |
|  | MDP0000579882 | homeobox-leucine zipper protein hat22-like | 20 | 0.00% | 71.40% | C:nucleus; F:DNA binding; F:nucleic acid binding transcription factor activity; P:cellular nitrogen compound metabolic process; P:biosynthetic process; C:protein complex; C:intracellular | - |
|  | MDP0000607885 | ras-related protein rabh1e-like | 2.00E+01 | 4.22E-35 | 96.65% | P:signal transduction; C:mitochondrion; F:ion binding; P:transport | - |
|  | MDP0000613004 | protein ralf-like 24 | 2.00E+01 | 5.22E-98 | 78.05% | - |  |
|  | MDP0000613011 | heat stress transcription factor a-5-like | 20 | 0.00% | 84.45% | C:nucleus; F:DNA binding; F:nucleic acid binding transcription factor activity; P:cellular nitrogen compound metabolic process; P:biosynthetic process; P:response to stress | - |
|  | MDP0000682858 | (-)-isopiperitenol (-)-carveol mitochondrial-like | 20 | 9.90E-55 | 79.10% | P:biological_process; F:oxidoreductase activity | - |
|  | MDP0000704869 | asparagine synthetase | 2.00E+01 | 9.31E-145 | 87.40% | F:ion binding; C:cytosol; P:cellular amino acid metabolic process; P:cellular nitrogen compound metabolic process; P:biosynthetic process; F:ligase activity | EC:6.3.5.4; EC:6.3.1; EC:6.3.1.1 |
|  | MDP0000716576 | vacuolar protein sorting-associated protein 32 homolog 2-like | 2.00E+01 | 1.15E-82 | 93.45% | P:transport; C:plasma membrane | - |
|  | MDP0000721663 | PREDICTED: uncharacterized protein LOC103410688 isoform X1 | 20 | 6.80E-153 | 80.85% | - |  |
|  | MDP0000722046 | glucose-1-phosphate adenylyltransferase small chloroplastic amyloplastic | 2.00E+01 | 0.00% | 93.25% | P:carbohydrate metabolic process; F:nucleotidyltransferase activity; P:generation of precursor metabolites and energy; C:cytoplasm; P:anatomical structure development; C:protein complex; P:biosynthetic process; C:extracellular region; C:plastid | EC:2.7.7.27 |
|  | MDP0000743523 | glutamate receptor -like | 10 | 7.74E-33 | 86.50% | - |  |
|  | MDP0000752179 | transmembrane 9 superfamily member 4 | 20 | 0.00% | 90.05% | C:endosome; C:cytoplasm; C:Golgi apparatus; C:vacuole; C:cellular_component | - |
|  | MDP0000763250 | asparagine synthetase | 2.00E+01 | 1.23E-53 | 83.40% | F:DNA binding; P:biological_process; P:sulfur compound metabolic process; F:ion binding; C:cytosol; P:cellular amino acid metabolic process; P:cellular nitrogen compound metabolic process; P:biosynthetic process; C:cellular_component; F:ligase activity; P:response to stress | EC:6.3.5.4; EC:6.3.1; EC:6.3.1.1 |
|  | MDP0000841002 | zinc finger ccch domain-containing protein 20-like | 20 | 0.00% | 74.70% | F:ion binding | - |
|  | MDP0000844309 | transmembrane 9 superfamily member 4 | 20 | 0.00% | 95.75% | C:endosome; C:cytoplasm; C:Golgi apparatus; C:vacuole; C:cellular_component | - |
|  | MDP0000884993 | glucose-1-phosphate adenylyltransferase small chloroplastic amyloplastic | 20 | 0.00% | 95.25% | P:carbohydrate metabolic process; F:nucleotidyltransferase activity; P:generation of precursor metabolites and energy; C:cytoplasm; P:anatomical structure development; C:protein complex; P:biosynthetic process; C:extracellular region; C:plastid | EC:2.7.7.27 |
|  | MDP0000938906 | ---NA--- | 0 |  |  | - |  |
| Variety Type | MDP0000122847 | 9-cis-epoxycarotenoid dioxygenase | 2.00E+01 | 5.72E-34 | 90.70% | C:chloroplast thylakoid membrane; F:9-cis-epoxycarotenoid dioxygenase activity; P:response to water deprivation; P:seed dormancy process; P:oxidation-reduction process | EC:1.13.11; EC:1.13.11.51 |
|  | MDP0000123712 | PREDICTED: uncharacterized protein LOC104900177 | 2000.00% | 2.73E-06 | 49.30% | - |  |
|  | MDP0000123768 | abc transporter b family member 11-like | 2000.00% | 0.00% | 87.45% | C:integral component of membrane; F:ATP binding; F:ATPase activity, coupled to transmembrane movement of substances; P:ATP catabolic process; P:transmembrane transport | EC:3.6.1; EC:3.6.1.3; EC:3.6.1.15 |
|  | MDP0000130493 | translation factor guf1 mitochondrial-like | 20 | 6.03E-158 | 72.45% | - |  |
|  | MDP0000131617 | | 1 | 0 | 100% | F:nucleotide binding; F:DNA-directed DNA polymerase activity; F:nucleoside binding; F:DNA binding; P:DNA replication initiation | EC:2.7.7.7 |
|  | MDP0000132257 | protein ralf-like 34 | 20 | 2.34E-64 | 81.15% | - |  |
|  | MDP0000135062 | PREDICTED: uncharacterized protein LOC103441514 | 2.00E+01 | 1.65E-149 | 57.75% | - |  |
|  | MDP0000135063 | homeobox protein knotted-1-like 3 isoform x2 | 2.00E+01 | 0.00% | 92.45% | C:nucleus; F:sequence-specific DNA binding transcription factor activity; F:sequence-specific DNA binding; P:regulation of transcription, DNA-templated; C:transcription factor complex; P:regulation of transcription, DNA-templated | - |
|  | MDP0000135177 | formin-like protein 14 | 20 | 2.89E-24 | 67.90% | - |  |
|  | MDP0000135249 | disease resistance protein rga3 | 2.00E+01 | 0.00% | 80.85% | F:ADP binding; F:protein binding | - |
|  | MDP0000135386 | s-adenosyl-l-methionine-dependent methyltransferases superfamily protein | 20 | 1.77E-15 | 90.45% | F:methyltransferase activity; P:rRNA methylation | - |
|  | MDP0000136517 | probable serine threonine-protein kinase at1g01540 | 20 | 0 | 84.75% | F:MAP kinase kinase kinase activity; F:ATP binding; P:activation of MAPKK activity; P:transmembrane receptor protein serine/threonine kinase signaling pathway; P:serine family amino acid metabolic process | EC:2.7.11.25; EC:2.7.11 |
|  | MDP0000136519 | subtilisin-like protease | 20 | 2.98E-60 | 68.30% | F:serine-type endopeptidase activity; P:proteolysis | EC:3.4.21 |
|  | MDP0000136520 | subtilisin-like protease | 20 | 6.34E-108 | 81.85% | F:serine-type endopeptidase activity; P:proteolysis | EC:3.4.21 |
|  | MDP0000137177 | cation calcium exchanger 2-like | 2000.00% | 0.00% | 81.85% | C:integral component of membrane; P:transmembrane transport | - |
|  | MDP0000137179 | protein pns1-like | 20 | 0 | 79.70% | C:integral component of membrane | - |
|  | MDP0000137181 | c2 domain-containing family protein | 2.00E+01 | 0.00% | 88.45% | F:protein binding | - |
|  | MDP0000140854 | receptor-like protein 12 | 20 | 1.73E-112 | 78.50% | - |  |
|  | MDP0000143114 | ---NA--- | 0.00% |  |  | - |  |
|  | MDP0000143130 | uncharacterized acetyltransferase at3g50280-like | 20 | 0 | 86.40% | F:transferase activity, transferring acyl groups other than amino-acyl groups; P:metabolic process | - |
|  | MDP0000156152 | protein far1-related sequence 5-like | 20 | 3.07E-22 | 63.90% | F:organic cyclic compound binding; F:heterocyclic compound binding | - |
|  | MDP0000158507 | nigrin b-like | 2.00E+01 | 0.00% | 67.15% | F:rRNA N-glycosylase activity; P:negative regulation of translation | EC:3.2.2.22 |
|  | MDP0000159264 | pentatricopeptide repeat-containing protein at5g66520-like | 2000.00% | 1.14E-31 | 76.45% | - |  |
|  | MDP0000160601 | heterogeneous nuclear ribonucleoprotein 1 | 2000.00% | 0.00% | 64.85% | F:nucleic acid binding; F:nucleotide binding | - |
|  | MDP0000161197 | embryonic protein dc-8-like | 2000.00% | 6.16E-109 | 70.70% | P:embryo development ending in seed dormancy; C:cellular_component | |
|  | MDP0000164429 | 3-phosphoshikimate 1-carboxyvinyltransferase 2 | 2.00E+01 | 0.00% | 89.35% | C:chloroplast stroma; F:3-phosphoshikimate 1-carboxyvinyltransferase activity; P:chorismate biosynthetic process; P:response to herbicide; P:tryptophan biosynthetic process; P:tyrosine biosynthetic process; P:L-phenylalanine biosynthetic process | EC:2.5.1.19 |
|  | MDP0000173824 | coiled-coil domain-containing protein | 2.00E+01 | 2.16E-45 | 65.60% | - |  |
|  | MDP0000174414 | subtilisin-like protease | 20 | 0 | 84.90% | F:serine-type endopeptidase activity; F:2-alkenal reductase [NAD(P)] activity; P:proteolysis; P:oxidation-reduction process | EC:3.4.21; EC:1.3.1.74 |
|  | MDP0000176158 | PREDICTED: uncharacterized protein LOC103423163 | 2000.00% | 0.00% | 84.35% | - |  |
|  | MDP0000178796 | glycerol kinase-like | 20 | 0 | 94.00% | F:glycerol kinase activity; P:response to molecule of bacterial origin; P:carbohydrate metabolic process; P:glycerol-3-phosphate metabolic process; P:response to microbial phytotoxin; P:phosphorylation; P:defense response to bacterium; P:response to karrikin; P:glycerolipid metabolic process | EC:2.7.1.30 |
|  | MDP0000178799 | nadh dehydrogenase | 2000.00% | 2.35E-180 | 95.45% | C:mitochondrial respiratory chain complex I; F:zinc ion binding; F:oxidoreductase activity; F:2 iron, 2 sulfur cluster binding; P:response to oxidative stress; P:oxidation-reduction process | - |
|  | MDP0000179991 | tmv resistance protein n-like isoform x2 | 20 | 5.03E-26 | 61.85% | - |  |
|  | MDP0000183373 | potassium channel skor | 20 | 5.88E-166 | 91.15% | F:outward rectifier potassium channel activity; P:potassium ion transmembrane transport; C:voltage-gated potassium channel complex; F:protein binding | - |
|  | MDP0000183375 | embryonic protein dc-8-like | 20 | 8.65E-90 | 66.95% | - |  |
|  | MDP0000183836 | embryonic protein dc-8-like | 2.00E+01 | 0.00% | 69.95% | P:embryo development ending in seed dormancy; C:cellular_component | |
|  | MDP0000184339 | uncharacterized acetyltransferase at3g50280-like | 20 | 7.46E-105 | 87.95% | C:cytosol; F:transferase activity, transferring acyl groups other than amino-acyl groups; P:metabolic process | - |
|  | MDP0000185314 | PREDICTED: uncharacterized protein LOC103446519 | 9 | 8.39E-27 | 63.22% | - |  |
|  | MDP0000185315 | probable s-adenosylmethionine-dependent methyltransferase at5g38100 | 20 | 4.27E-36 | 70.35% | F:methyltransferase activity | - |
|  | MDP0000186418 | mannosyl-oligosaccharide glucosidase gcs1-like | 2.00E+01 | 1.59E-162 | 89.25% | F:alpha-1,4-glucosidase activity; F:mannosyl-oligosaccharide glucosidase activity; P:epidermal cell differentiation; P:root epidermal cell differentiation; P:starch metabolic process; P:sucrose metabolic process; P:galactose metabolic process; C:glucosidase II complex | EC:3.2.1.106; EC:3.2.1.20; EC:3.2.1 |
|  | MDP0000191641 | wd-repeat isoform partial | 20 | 4.06E-93 | 83.45% | - |  |
|  | MDP0000192054 | heterogeneous nuclear ribonucleoprotein 1-like | 20 | 0 | 79.15% | F:nucleic acid binding; F:nucleotide binding | - |
|  | MDP0000192819 | soluble diacylglycerol acyltransferase | 20 | 0 | 71.20% | F:diacylglycerol O-acyltransferase activity; P:triglyceride biosynthetic process; C:cytosol; F:transferase activity; P:metabolic process; F:transferase activity, transferring acyl groups | |
|  | MDP0000195139 | disease resistance protein rga3 | 2000.00% | 0.00% | 80.35% | F:protein binding; F:ADP binding | - |
|  | MDP0000195887 | probable polyribonucleotide nucleotidyltransferase chloroplastic | 20 | 4.70E-48 | 68.80% | F:transferase activity; P:RNA metabolic process | - |
|  | MDP0000195987 | probable receptor protein kinase tmk1 | 20 | 1.65E-49 | 71.85% | C:membrane; F:nucleotide binding; F:protein serine/threonine kinase activity; P:signal transduction; P:phosphorylation; P:serine family amino acid metabolic process | EC:2.7.11 |
|  | MDP0000196140 | PREDICTED: uncharacterized protein LOC103410720 isoform X2 | 2.00E+01 | 0.00% | 88.95% | C:plasma membrane | - |
|  | MDP0000197246 | dna-directed rna polymerases iv and v subunit 6a-like | 2.00E+01 | 3.68E-59 | 77.25% | C:DNA-directed RNA polymerase IV complex; C:DNA-directed RNA polymerase V complex; C:DNA-directed RNA polymerase II, core complex; F:DNA binding; F:DNA-directed RNA polymerase activity; P:transcription, DNA-templated; C:nucleolus; P:purine nucleobase metabolic process; P:pyrimidine nucleobase metabolic process | EC:2.7.7.6 |
|  | MDP0000197247 | phosphatidylinositol n-acetylglucosaminyltransferase subunit c | 20 | 3.79E-129 | 89.45% | C:integral component of membrane; F:phosphatidylinositol N-acetylglucosaminyltransferase activity; P:GPI anchor biosynthetic process; P:pollen germination; P:pollen tube growth | EC:2.4.1; EC:2.4.1.198 |
|  | MDP0000198805 | protein kinase pinoid | 20 | 1.51E-57 | 69.05% | C:cell surface; F:protein serine/threonine kinase activity; F:identical protein binding; P:response to light stimulus; P:auxin-activated signaling pathway; P:auxin polar transport; P:positive gravitropism; P:root hair initiation; P:root hair elongation; P:cotyledon development; P:response to karrikin; P:serine family amino acid metabolic process; F:ATP binding; P:protein phosphorylation | EC:2.7.11 |
|  | MDP0000202018 | probable receptor protein kinase tmk1 | 20 | 3.87E-42 | 96.80% | C:plasma membrane; C:integral component of membrane; F:transmembrane receptor protein serine/threonine kinase activity; F:ATP binding; P:protein phosphorylation; P:transmembrane receptor protein serine/threonine kinase signaling pathway; P:serine family amino acid metabolic process | EC:2.7.11 |
|  | MDP0000203903 | probable polyribonucleotide nucleotidyltransferase chloroplastic | 20 | 0 | 88.60% | C:chloroplast stroma; F:3'-5'-exoribonuclease activity; F:RNA binding; F:polyribonucleotide nucleotidyltransferase activity; P:mRNA catabolic process; P:negative regulation of isopentenyl diphosphate biosynthetic process, methylerythritol 4-phosphate pathway; P:chlorophyll biosynthetic process; P:cellular response to phosphate starvation; P:carotene biosynthetic process; P:xanthophyll biosynthetic process; P:chloroplast RNA processing; P:RNA phosphodiester bond hydrolysis, exonucleolytic; P:regulation of RNA metabolic process; P:purine nucleobase metabolic process; P:pyrimidine nucleobase metabolic process | EC:3.1; EC:3.1.13; EC:3.1.15; EC:2.7.7.8 |
|  | MDP0000209135 | heat stress transcription factor b-4 | 2.00E+01 | 0.00% | 74.75% | C:nucleus; F:sequence-specific DNA binding transcription factor activity; F:sequence-specific DNA binding; P:regulation of transcription, DNA-templated; P:response to heat; C:transcription factor complex; P:regulation of transcription, DNA-templated | - |
|  | MDP0000209670 | | 100.00% | 0.00% | 100% | F:protein binding; P:regulation of transcription, DNA-templated | - |
|  | MDP0000209674 | haloacid dehalogenase-like hydrolase domain-containing protein 3 | 2000.00% | 1.65E-38 | 91.15% | F:hydrolase activity; P:metabolic process | - |
|  | MDP0000219303 | transcription factor hec2-like | 20 | 1.32E-98 | 68.55% | F:protein dimerization activity; P:gynoecium development | - |
|  | MDP0000230533 | PREDICTED: uncharacterized protein LOC103441111 | 2000.00% | 0.00% | 88.75% | C:plasma membrane | - |
|  | MDP0000238968 | protein chloroplastic | 20 | 0 | 70.75% | - |  |
|  | MDP0000243236 | cation calcium exchanger 2-like | 2000.00% | 0.00% | 76.60% | C:integral component of membrane; P:transmembrane transport | - |
|  | MDP0000243380 | | 100.00% | 0.00% | 100% | F:protein binding | - |
|  | MDP0000245028 | probable serine threonine-protein kinase at1g01540 | 20 | 0 | 86.35% | F:protein serine/threonine kinase activity; F:non-membrane spanning protein tyrosine kinase activity; F:ATP binding; P:peptidyl-tyrosine phosphorylation; P:serine family amino acid metabolic process | EC:2.7.10; EC:2.7.11; EC:2.7.10.2 |
|  | MDP0000249205 | proline-rich protein 12-like | 20 | 5.26E-31 | 64.55% | - |  |
|  | MDP0000249209 | xylosyltransferase 1-like | 20 | 0 | 89.75% | C:membrane; F:acetylglucosaminyltransferase activity; P:metabolic process; P:double fertilization forming a zygote and endosperm; P:pollen tube development | EC:2.4.1 |
|  | MDP0000249539 | disease resistance protein rga3 | 2.00E+01 | 0.00% | 83.25% | F:protein binding; F:ADP binding | - |
|  | MDP0000249581 | dna-directed rna polymerase subunit beta | 2.00E+01 | 0.00% | 82.95% | - |  |
|  | MDP0000254312 | potassium channel skor | 20 | 0 | 93.20% | F:outward rectifier potassium channel activity; P:potassium ion transmembrane transport; C:voltage-gated potassium channel complex; F:protein binding | - |
|  | MDP0000255896 | probable sucrose-phosphate synthase 4 | 20 | 4.38E-14 | 93.10% | C:plasma membrane; F:sucrose-phosphate synthase activity; P:sucrose metabolic process; P:biosynthetic process; P:starch metabolic process | EC:2.4.1.14; EC:2.4.1 |
|  | MDP0000258367 | ---NA--- | 0.00E+00 |  |  | F:phosphoric diester hydrolase activity; P:lipid metabolic process | EC:3.1 |
|  | MDP0000268468 | protein root initiation defective 3 | 20 | 2.33E-35 | 51.10% | F:protein binding | - |
|  | MDP0000269048 | maltase- intestinal | 2000.00% | 5.18E-105 | 77.30% | - |  |
|  | MDP0000273271 | disease resistance protein rga3 | 2000.00% | 1.19E-144 | 82.90% | F:protein binding | - |
|  | MDP0000274556 | probable mitochondrial-processing peptidase subunit beta | 20 | 1.14E-71 | 92.65% | F:metalloendopeptidase activity; F:metal ion binding; P:proteolysis | EC:3.4.24 |
|  | MDP0000275168 | PREDICTED: uncharacterized protein LOC103407361 | 2.00E+01 | 1.17E-102 | 54.00% | F:zinc ion binding | - |
|  | MDP0000275789 | protein srg1-like | 20 | 1.01E-68 | 69.70% | F:oxidoreductase activity, acting on paired donors, with incorporation or reduction of molecular oxygen, 2-oxoglutarate as one donor, and incorporation of one atom each of oxygen into both donors | EC:1.14.11 |
|  | MDP0000276022 | PREDICTED: uncharacterized protein LOC103401439 | 2.00E+01 | 0.00% | 86.30% | F:triglyceride lipase activity; P:lipid catabolic process; P:glycerolipid metabolic process | EC:3.1.1; EC:3.1.1.1; EC:3.1.1.3 |
|  | MDP0000278543 | 50s ribosomal protein l25-like | 2000.00% | 8.99E-175 | 88.00% | C:ribosome; F:structural constituent of ribosome; F:5S rRNA binding; P:translation; P:ribosome biogenesis; F:protein binding; P:signal transduction | - |
|  | MDP0000280551 | pyrophosphate-energized vacuolar membrane proton pump-like | 20 | 0 | 96.80% | C:membrane; F:inorganic diphosphatase activity; F:hydrogen-translocating pyrophosphatase activity; P:proton transport; P:transmembrane transport; P:oxidative phosphorylation | EC:3.6.1.1; EC:3.6.1 |
|  | MDP0000281041 | potassium channel skor-like isoform x2 | 20 | 8.17E-65 | 88.05% | F:outward rectifier potassium channel activity; P:potassium ion transmembrane transport; C:voltage-gated potassium channel complex | - |
|  | MDP0000281449 | alpha beta-hydrolases superfamily | 2000.00% | 0.00% | 82.80% | F:triglyceride lipase activity; P:lipid catabolic process; P:glycerolipid metabolic process | EC:3.1.1; EC:3.1.1.1; EC:3.1.1.3 |
|  | MDP0000282711 | pra1 family protein f4-like | 20 | 2.82E-90 | 70.80% | - |  |
|  | MDP0000283955 | pentatricopeptide repeat-containing protein at1g26500 | 2000.00% | 0.00% | 85.40% | - |  |
|  | MDP0000283985 | ---NA--- | 0.00E+00 |  |  | F:RNA binding; F:nucleotide binding | - |
|  | MDP0000284296 | nad -binding rossmann-fold superfamily protein isoform 1 | 2.00E+01 | 0.00% | 84.35% | F:catalytic activity; P:metabolic process | - |
|  | MDP0000284298 | probable receptor protein kinase tmk1 | 20 | 2.28E-93 | 69.70% | F:ATP binding; P:protein phosphorylation; F:protein serine/threonine kinase activity | EC:2.7.11 |
|  | MDP0000290186 | disease resistance protein rga3 | 2.00E+01 | 0.00% | 78.85% | F:ADP binding; F:protein binding | - |
|  | MDP0000291076 | 60s ribosomal protein l19-1 | 2.00E+01 | 8.33E-114 | 97.75% | C:nucleolus; C:plasma membrane; C:plasmodesma; C:cytosolic large ribosomal subunit; F:structural constituent of ribosome; P:translation; P:ribosome biogenesis | - |
|  | MDP0000291077 | proline--trna ligase | 20 | 0 | 93.30% | C:cytosol; C:plasmodesma; C:membrane; F:proline-tRNA ligase activity; F:ATP binding; P:prolyl-tRNA aminoacylation; P:arginine metabolic process; P:proline metabolic process | EC:6.1.1; EC:6.1.1.15 |
|  | MDP0000291386 | phd finger protein male meiocyte death 1 | 20 | 0 | 76.80% | F:zinc ion binding; P:reproduction; P:single-organism cellular process; F:protein binding | - |
|  | MDP0000291387 | potassium channel skor-like isoform x3 | 20 | 6.95E-42 | 86.35% | F:outward rectifier potassium channel activity; P:potassium ion transmembrane transport; C:voltage-gated potassium channel complex | - |
|  | MDP0000292500 | probable polyribonucleotide nucleotidyltransferase chloroplastic | 20 | 0 | 87.55% | C:chloroplast stroma; F:3'-5'-exoribonuclease activity; F:RNA binding; F:polyribonucleotide nucleotidyltransferase activity; P:mRNA catabolic process; P:negative regulation of isopentenyl diphosphate biosynthetic process, methylerythritol 4-phosphate pathway; P:chlorophyll biosynthetic process; P:cellular response to phosphate starvation; P:carotene biosynthetic process; P:xanthophyll biosynthetic process; P:chloroplast RNA processing; P:RNA phosphodiester bond hydrolysis, exonucleolytic; P:regulation of RNA metabolic process; P:purine nucleobase metabolic process; P:pyrimidine nucleobase metabolic process | EC:3.1; EC:3.1.13; EC:3.1.15; EC:2.7.7.8 |
|  | MDP0000292776 | wd-repeat isoform partial | 20 | 2.88E-44 | 56.85% | F:protein binding | - |
|  | MDP0000293040 | replication factor c subunit 3 | 20 | 0 | 96.70% | F:nucleotide binding; F:DNA binding; F:nucleoside-triphosphatase activity; P:DNA replication | EC:3.6.1; EC:3.6.1.15 |
|  | MDP0000294613 | ---NA--- | 0.00E+00 |  |  | F:transferase activity, transferring acyl groups other than amino-acyl groups | - |
|  | MDP0000294614 | uncharacterized acetyltransferase at3g50280-like | 20 | 0 | 80.30% | F:transferase activity, transferring acyl groups other than amino-acyl groups; F:DNA binding; P:regulation of transcription, DNA-templated | - |
|  | MDP0000297288 | trafficking protein particle complex subunit 9 isoform x1 | 20 | 9.50E-125 | 88.90% | - |  |
|  | MDP0000298555 | protein gamete expressed 1 | 20 | 0 | 80.55% | - |  |
|  | MDP0000303781 | f-box lrr-repeat protein 13-like isoform x2 | 20 | 9.39E-134 | 80.15% | - |  |
|  | MDP0000303782 | cobw domain-containing protein 1-like | 2.00E+01 | 0.00% | 84.70% | C:plastid chromosome; C:chloroplast stroma | - |
|  | MDP0000304326 | triphosphate tunel metalloenzyme 3-like | 20 | 1.15E-130 | 85.45% | - |  |
|  | MDP0000304327 | nbs-lrr disease resistance protein | 2.00E+01 | 0.00% | 79.95% | F:ADP binding; F:diacylglycerol kinase activity; P:protein kinase C-activating G-protein coupled receptor signaling pathway; P:metabolic process; F:NAD+ kinase activity | EC:2.7.1.23; EC:2.7.1.107 |
|  | MDP0000304601 | disease resistance protein rga3 | 2000.00% | 0.00% | 80.85% | F:protein binding; F:ADP binding | - |
|  | MDP0000310375 | maltase- intestinal | 2.00E+01 | 0.00% | 83.10% | F:zinc ion binding | |
|  | MDP0000310376 | cationic amino acid transporter vacuolar-like | 2.00E+01 | 0.00% | 90.95% | C:plant-type vacuole membrane; C:integral component of membrane; F:amino acid transmembrane transporter activity; P:amino acid transmembrane transport | - |
|  | MDP0000315270 | ankyrin repeat-containing protein at3g12360-like | 2000.00% | 1.02E-145 | 84.75% | F:coenzyme binding; P:coenzyme A metabolic process; F:hydroxymethylglutaryl-CoA reductase (NADPH) activity; P:oxidation-reduction process; F:protein dimerization activity; P:transcription, DNA-templated | EC:1.1.1.34 |
|  | MDP0000320739 | | 100.00% | 0.00% | 100% | F:protein dimerization activity | - |
|  | MDP0000322089 | PREDICTED: uncharacterized protein LOC103324616 | 2.00E+01 | 0.00% | 62.60% | F:nucleic acid binding | - |
|  | MDP0000328704 | ---NA--- | 0.00% |  |  | - |  |
|  | MDP0000331298 | hsp40 cysteine-rich domain superfamily protein isoform 1 | 2.00E+01 | 6.00E-87 | 83.65% | F:heat shock protein binding; F:unfolded protein binding | - |
|  | MDP0000338651 | protein unc-13-c-like protein | 20 | 1.10E-18 | 94.55% | F:molecular_function; P:biological_process; C:cellular_component | |
|  | MDP0000343581 | ---NA--- | 0.00% |  |  | - |  |
|  | MDP0000359637 | ---NA--- | 0.00% |  |  | - |  |
|  | MDP0000360924 | ---NA--- | 0.00E+00 |  |  | - |  |
|  | MDP0000361342 | pollen-specific leucine-rich repeat extensin-like protein 1 | 13 | 4.58E-29 | 70.92% | - |  |
|  | MDP0000389293 | probable adp-ribosylation factor gtpase-activating protein agd6 | 20 | 1.81E-19 | 94.15% | C:nucleus; C:cytosol; F:ARF GTPase activator activity; F:zinc ion binding; F:transaminase activity; P:regulation of ARF GTPase activity; P:positive regulation of GTPase activity | - |
|  | MDP0000439832 | uncharacterized oxidoreductase at4g09670-like | 20 | 0 | 85.25% | F:oxidoreductase activity; P:oxidation-reduction process | - |
|  | MDP0000440151 | potassium channel skor-like isoform x2 | 20 | 2.47E-39 | 87.90% | F:outward rectifier potassium channel activity; P:potassium ion transmembrane transport; C:voltage-gated potassium channel complex | - |
|  | MDP0000445632 | 28s ribosomal protein mitochondrial | 2.00E+01 | 6.48E-47 | 81.60% | C:ribosome; F:structural constituent of ribosome; P:translation; P:ribosome biogenesis | - |
|  | MDP0000469664 | lrr receptor-like serine threonine-protein kinase rch1 | 2000.00% | 0.00% | 85.65% | C:integral component of membrane; F:protein kinase activity; F:ATP binding; P:protein phosphorylation; F:protein binding | - |
|  | MDP0000512999 | glycine dehydrogenase mitochondrial | 20 | 4.77E-40 | 84.95% | C:mitochondrion; C:chloroplast thylakoid; C:chloroplast stroma; C:chloroplast envelope; C:apoplast; F:glycine dehydrogenase (decarboxylating) activity; F:ATP binding; F:pyridoxal phosphate binding; P:glycine catabolic process; P:response to cadmium ion; P:oxidation-reduction process; P:L-serine metabolic process; P:threonine metabolic process; P:biosynthetic process | EC:1.4.4.2 |
|  | MDP0000514448 | vq motif-containing | 20 | 1.54E-118 | 65.95% | - |  |
|  | MDP0000516523 | ---NA--- | 0.00% |  |  | - |  |
|  | MDP0000519674 | subtilisin-like protease | 20 | 8.55E-90 | 70.50% | P:proteolysis; F:serine-type endopeptidase activity | EC:3.4.21 |
|  | MDP0000523812 | transcription factor spatula-like isoform x1 | 20 | 6.22E-132 | 79.90% | F:protein dimerization activity | - |
|  | MDP0000527046 | esterase vc_a0580-like | 2000.00% | 5.08E-91 | 88.85% | C:peroxisome; P:phylloquinone biosynthetic process | - |
|  | MDP0000531724 | phosphate transporter pho1 homolog 10-like | 20 | 3.46E-61 | 67.25% | C:integral component of membrane; P:protein phosphorylation; F:protein kinase activity; F:ATP binding | - |
|  | MDP0000572169 | zinc finger protein 4-like | 20 | 1.54E-37 | 68.90% | F:metal ion binding | - |
|  | MDP0000577338 | PREDICTED: uncharacterized protein LOC103423750 | 2.00E+01 | 1.17E-66 | 73.05% | - |  |
|  | MDP0000636876 | tmv resistance protein n-like | 20 | 0 | 79.90% | F:ADP binding; P:defense response; P:signal transduction; F:protein binding | - |
|  | MDP0000650075 | probable boi-related e3 ubiquitin-protein ligase 2 | 20 | 6.20E-176 | 82.00% | F:zinc ion binding; F:protein binding | - |
|  | MDP0000652388 | cation calcium exchanger 1-like | 2.00E+01 | 0.00% | 80.20% | C:integral component of membrane; P:transmembrane transport | - |
|  | MDP0000711911 | nigrin b-like | 2000.00% | 0.00% | 65.75% | F:rRNA N-glycosylase activity; P:negative regulation of translation | EC:3.2.2.22 |
|  | MDP0000713910 | transcription factor bhlh95 | 20 | 2.28E-168 | 70.40% | F:protein dimerization activity | - |
|  | MDP0000717184 | protein suppressor of npr1- constitutive 1-like | 20 | 9.07E-163 | 78.35% | F:protein binding | - |
|  | MDP0000717791 | probable boi-related e3 ubiquitin-protein ligase 2 | 20 | 6.18E-178 | 83.95% | F:zinc ion binding; F:protein binding | - |
|  | MDP0000729521 | uncharacterized acetyltransferase at3g50280-like | 20 | 0 | 81.25% | F:transferase activity, transferring acyl groups other than amino-acyl groups; P:metabolic process | - |
|  | MDP0000735372 | magnesium-protoporphyrin ix monomethyl ester | 2.00E+01 | 2.85E-135 | 91.85% | C:chloroplast thylakoid; C:chloroplast envelope; F:metal ion binding; F:magnesium-protoporphyrin IX monomethyl ester (oxidative) cyclase activity; P:chloroplast organization; P:photosynthesis; P:chlorophyll biosynthetic process; P:oxidation-reduction process; P:regulation of tetrapyrrole metabolic process | EC:1.14.13.81; EC:1.14.13 |
|  | MDP0000745770 | u-box domain-containing protein 21-like | 20 | 7.62E-82 | 78.50% | C:ubiquitin ligase complex; F:ubiquitin-protein transferase activity; P:protein ubiquitination | - |
|  | MDP0000747281 | btb poz and math domain-containing protein 4-like | 2000.00% | 0.00% | 91.70% | C:cytosol; P:cellular response to water deprivation; P:cellular response to salt stress; F:protein binding | - |
|  | MDP0000753788 | 40s ribosomal protein s13 | 2.00E+01 | 7.64E-66 | 97.80% | C:nucleolus; C:endoplasmic reticulum; C:ribosome; C:membrane; F:structural constituent of ribosome; P:cytokinesis by cell plate formation; P:translation; P:leaf morphogenesis; P:trichome morphogenesis; P:ribosome biogenesis | - |
|  | MDP0000760132 | pentatricopeptide repeat-containing protein at1g26500 | 2000.00% | 0.00% | 85.60% | - |  |
|  | MDP0000775126 | PREDICTED: pinin-like | 9.00E+00 | 1.73E-39 | 80.67% | - |  |
|  | MDP0000784090 | tmv resistance protein n-like | 20 | 4.04E-19 | 54.60% | - |  |
|  | MDP0000784168 | mitogen-activated protein kinase kinase kinase 3-like | 2.00E+01 | 0.00% | 79.65% | F:protein serine/threonine kinase activity; F:ATP binding; P:protein phosphorylation; P:serine family amino acid metabolic process | EC:2.7.11 |
|  | MDP0000806017 | ---NA--- | 0.00% |  |  | - |  |
|  | MDP0000810351 | disease resistance protein rga3 | 20 | 0.00% | 81.05% | F:protein binding; F:ADP binding | - |
|  | MDP0000818448 | disease resistance protein rga3 | 2000.00% | 1.87E-142 | 80.85% | F:protein binding | - |
|  | MDP0000835932 | mitogen-activated protein kinase kinase kinase 3-like | 2.00E+01 | 0.00% | 79.65% | F:protein serine/threonine kinase activity; F:ATP binding; P:protein phosphorylation; P:serine family amino acid metabolic process | EC:2.7.11 |
|  | MDP0000848029 | protein root initiation defective 3-like | 20 | 1.37E-38 | 79.80% | - |  |
|  | MDP0000851135 | pentatricopeptide repeat-containing protein at5g66520-like | 20 | 6.94E-28 | 78.55% | - |  |
|  | MDP0000853127 | arogenate dehydrogenase chloroplastic-like | 2.00E+01 | 0.00% | 88.20% | F:prephenate dehydrogenase (NADP+) activity; F:prephenate dehydrogenase activity; P:tyrosine biosynthetic process; P:oxidation-reduction process; P:tryptophan biosynthetic process; P:L-phenylalanine biosynthetic process | EC:1.3.1.12; EC:1.3.1.13 |
|  | MDP0000857821 | protein chloroplastic | 20 | 1.75E-51 | 93.95% | - |  |
|  | MDP0000867534 | PREDICTED: uncharacterized protein LOC103950428 | 2.00E+01 | 6.98E-52 | 77.40% | - |  |
|  | MDP0000869168 | pi-plc x domain-containing protein at5g67130-like | 20 | 7.31E-96 | 82.05% | F:phosphoric diester hydrolase activity; P:lipid metabolic process | EC:3.1 |
|  | MDP0000879254 | replication factor c subunit 3 | 20 | 1.06E-111 | 97.10% | F:nucleotide binding; F:DNA binding; F:nucleoside-triphosphatase activity; P:DNA replication | EC:3.6.1; EC:3.6.1.15 |
|  | MDP0000879258 | splicing factor 3b subunit | 20 | 1.30E-23 | 95.60% | - |  |
|  | MDP0000893755 | cationic amino acid transporter vacuolar-like | 2000.00% | 0.00% | 90.85% | C:plant-type vacuole membrane; C:integral component of membrane; F:amino acid transmembrane transporter activity; P:amino acid transmembrane transport | - |
|  | MDP0000921094 | subtilisin-like protease | 20 | 0 | 80.70% | C:extracellular region; F:serine-type endopeptidase activity; P:proteolysis | EC:3.4.21 |
| Bitterness | MDP0000052541 | embryo defective 1381 isoform 1 | 20 | 0 | 90.35% | - |  |
|  | MDP0000119516 | 3-ketodihydrosphingosine reductase-like | 20 | 0 | 88.50% | F:oxidoreductase activity; P:metabolic process | - |
|  | MDP0000123824 | 26s proteasome non-atpase regulatory subunit 7 homolog a-like | 20 | 4.55E-10 | 58.85% | P:leaf morphogenesis; C:cytosol; P:embryo development ending in seed dormancy; C:proteasome complex | |
|  | MDP0000124900 | methionine aminopeptidase chloroplastic | 20 | 0 | 86.90% | P:proteolysis; F:aminopeptidase activity; F:metalloexopeptidase activity | EC:3.4.11 |
|  | MDP0000127054 | ethylene-responsive transcription factor erf003-like | 20 | 1.58E-101 | 84.35% | P:regulation of transcription, DNA-templated; F:sequence-specific DNA binding transcription factor activity; F:DNA binding; C:transcription factor complex; P:regulation of transcription, DNA-templated | - |
|  | MDP0000128281 | vacuolar protein 8 | 20 | 0 | 87.00% | F:protein binding | - |
|  | MDP0000129011 | transcription factor bhlh68-like isoform x1 | 20 | 0 | 77.40% | F:protein dimerization activity | - |
|  | MDP0000131356 | probable carboxylesterase 6 | 20 | 0 | 74.50% | P:metabolic process; F:hydrolase activity | - |
|  | MDP0000132527 | sucrose synthase | 20 | 0 | 88.30% | P:sucrose metabolic process; P:biosynthetic process | - |
|  | MDP0000134560 | probable histone-lysine n-methyltransferase atxr3 | 20 | 3.65E-64 | 80.25% | F:methyltransferase activity; P:methylation | - |
|  | MDP0000135679 | thiamine-repressible mitochondrial transport protein thi74-like | 20 | 0 | 78.10% | C:integral component of membrane | - |
|  | MDP0000135680 | probable monogalactosyldiacylglycerol chloroplastic | 20 | 0 | 87.65% | P:lipid glycosylation; F:carbohydrate binding; F:transferase activity, transferring hexosyl groups; P:glycolipid biosynthetic process | - |
|  | MDP0000136728 | methionine aminopeptidase chloroplastic | 20 | 0 | 86.70% | F:aminopeptidase activity; P:proteolysis; F:metalloexopeptidase activity | EC:3.4.11 |
|  | MDP0000139500 | probable histone-lysine n-methyltransferase atxr3 | 20 | 3.66E-64 | 80.25% | F:methyltransferase activity; P:methylation | - |
|  | MDP0000141005 | serine threonine-protein phosphatase 2a 65 kda regulatory subunit a beta isoform | 20 | 0 | 94.55% | F:binding | - |
|  | MDP0000145027 | tyrosine-protein phosphatase | 20 | 2.37E-158 | 86.40% | F:protein tyrosine phosphatase activity; P:protein dephosphorylation; P:tyrosine metabolic process | EC:3.1.3.16; EC:3.1; EC:3.1.3.48; EC:3.1.3.41 |
|  | MDP0000148855 | protein glutamine dumper 5-like | 20 | 2.18E-94 | 76.40% | - |  |
|  | MDP0000154158 | gdt1-like protein 4 | 20 | 4.03E-35 | 96.05% | C:membrane | - |
|  | MDP0000155087 | uncharacterized loc101206567 | 20 | 0 | 89.80% | C:endosome; C:trans-Golgi network | - |
|  | MDP0000155673 | spindle and kinetochore-associated 2 | 20 | 1.92E-75 | 90.05% | - |  |
|  | MDP0000155674 | dynamin-related protein 3a-like isoform x1 | 20 | 0 | 80.80% | F:GTPase activity; F:GTP binding | EC:3.6.1; EC:3.6.1.15 |
|  | MDP0000155675 | protein tic chloroplastic-like | 20 | 0 | 90.00% | F:chlorophyllide a oxygenase [overall] activity; P:oxidation-reduction process; F:2 iron, 2 sulfur cluster binding | EC:1.13.12; EC:1.14.13.122 |
|  | MDP0000157412 | ctl-like protein ddb_g0274487 | 20 | 0 | 88.70% | C:integral component of membrane | - |
|  | MDP0000159583 | PREDICTED: uncharacterized protein LOC103416009 | 20 | 2.32E-113 | 61.15% | - |  |
|  | MDP0000160232 | disease resistance protein rga3 | 20 | 0 | 78.55% | F:protein binding; F:ADP binding | - |
|  | MDP0000160621 | probable mitochondrial adenine nucleotide transporter btl3 | 20 | 1.71E-32 | 55.95% | C:integral component of membrane; C:membrane; P:transmembrane transport; P:transport | |
|  | MDP0000167338 | duf868 family protein | 20 | 0 | 73.80% | C:plasma membrane | |
|  | MDP0000167343 | probable flavin-containing monooxygenase 1 | 20 | 0 | 86.15% | F:NADP binding; F:flavin adenine dinucleotide binding; P:oxidation-reduction process; F:N,N-dimethylaniline monooxygenase activity | EC:1.14.13.8; EC:1.14.13 |
|  | MDP0000171928 | leucoanthocyanidin reductase-like | 20 | 2.52E-131 | 92.90% | F:leucoanthocyanidin reductase activity; P:oxidation-reduction process | EC:1.17.1; EC:1.17.1.3 |
|  | MDP0000171929 | leucoanthocyanidin reductase | 20 | 1.97E-22 | 83.60% | F:leucoanthocyanidin reductase activity; P:oxidation-reduction process | EC:1.17.1; EC:1.17.1.3 |
|  | MDP0000180004 | allene oxide cyclase chloroplastic-like | 20 | 2.11E-140 | 85.80% | F:isomerase activity; C:chloroplast | - |
|  | MDP0000180005 | methionine aminopeptidase chloroplastic-like | 20 | 0 | 87.90% | F:aminopeptidase activity; F:metalloexopeptidase activity; P:proteolysis | EC:3.4.11 |
|  | MDP0000181021 | ---NA--- | 0 |  |  | - |  |
|  | MDP0000181352 | cytochrome c-type biogenesis | 20 | 2.05E-103 | 91.40% | C:mitochondrial inner membrane; C:protein complex; F:oxidoreductase activity; P:embryo development; P:oxidation-reduction process | - |
|  | MDP0000182500 | cysteine-rich receptor-like protein kinase 10 | 20 | 0 | 68.00% | F:ATP binding; P:protein phosphorylation; F:protein serine/threonine kinase activity; P:serine family amino acid metabolic process | EC:2.7.11 |
|  | MDP0000188242 | dof zinc finger | 20 | 2.11E-96 | 70.50% | F:translation elongation factor activity; C:eukaryotic translation elongation factor 1 complex; F:DNA binding; P:regulation of transcription, DNA-templated; C:ribosome; P:regulation of translational elongation | - |
|  | MDP0000188336 | mediator of rna polymerase ii transcription subunit 25-like isoform x2 | 20 | 0 | 83.25% | P:signal transduction; P:response to external stimulus; P:response to red or far red light; P:regulation of flower development; P:positive regulation of biological process | - |
|  | MDP0000188337 | lysine histidine transporter-like 6 | 20 | 0 | 91.80% | C:integral component of membrane | - |
|  | MDP0000188338 | 40s ribosomal protein s3a-2-like | 20 | 8.33E-93 | 64.70% | F:structural constituent of ribosome; P:translation; C:ribosome; P:ribosome biogenesis | - |
|  | MDP0000190319 | protein transport protein sec24-like at4g32640 | 20 | 3.14E-59 | 67.70% | P:protein transport; P:intracellular transport | - |
|  | MDP0000193411 | cysteine-rich rlk isoform 1 | 20 | 1.18E-74 | 70.90% | P:protein phosphorylation; F:protein kinase activity; F:ATP binding | - |
|  | MDP0000198209 | leucine-rich repeat receptor-like serine threonine-protein kinase at2g14440 | 20 | 5.91E-71 | 61.85% | C:endosome; C:vacuole; C:trans-Golgi network; C:plasma membrane | - |
|  | MDP0000201211 | |  |  |  |  |  |
|  | MDP0000201494 | cysteine-rich receptor-like protein kinase 10 | 20 | 0 | 68.00% | F:ATP binding; P:protein phosphorylation; F:protein serine/threonine kinase activity; P:serine family amino acid metabolic process | EC:2.7.11 |
|  | MDP0000202669 | zinc finger protein constans-like 4 | 20 | 0 | 86.50% | F:zinc ion binding; C:intracellular; F:protein binding | - |
|  | MDP0000207420 | palmitoyl-monogalactosyldiacylglycerol delta-7 chloroplastic-like | 20 | 4.14E-81 | 83.50% | P:oxidation-reduction process; F:oxidoreductase activity, acting on paired donors, with oxidation of a pair of donors resulting in the reduction of molecular oxygen to two molecules of water | EC:1.14.19 |
|  | MDP0000207423 | vacuolar protein 8 | 20 | 0 | 87.10% | F:protein binding; C:vacuolar proton-transporting V-type ATPase, V1 domain; F:proton-transporting ATPase activity, rotational mechanism; P:ATP hydrolysis coupled proton transport; P:oxidative phosphorylation | EC:3.6.1; EC:3.6.1.3; EC:3.6.1.15 |
|  | MDP0000208044 | metallo-hydrolase oxidoreductase superfamily protein isoform 1 | 20 | 0 | 90.20% | C:chloroplast; F:hydrolase activity; P:metabolic process | - |
|  | MDP0000208045 | 40s ribosomal protein s3a-2-like | 20 | 6.72E-42 | 55.20% | C:ribonucleoprotein complex; C:intracellular non-membrane-bounded organelle; C:cytoplasmic part | - |
|  | MDP0000208046 | lysine histidine transporter-like 6 | 20 | 0 | 92.40% | C:integral component of membrane | - |
|  | MDP0000208228 | n-acylphosphatidylethanolamine synthase-like | 20 | 1.49E-77 | 65.25% | F:transferase activity, transferring acyl groups; P:phospholipid metabolic process | - |
|  | MDP0000215799 | serine threonine-protein kinase tor | 20 | 1.35E-49 | 78.95% | F:protein binding | - |
|  | MDP0000215801 | catalytic coenzyme binding protein | 20 | 0 | 85.85% | C:plasma membrane | - |
|  | MDP0000216289 | palmitoyl-monogalactosyldiacylglycerol delta-7 chloroplastic-like | 20 | 1.51E-95 | 89.45% | F:oxidoreductase activity, acting on paired donors, with oxidation of a pair of donors resulting in the reduction of molecular oxygen to two molecules of water; P:oxidation-reduction process; P:lipid metabolic process | EC:1.14.19 |
|  | MDP0000216620 | 40s ribosomal protein s3a-2-like | 20 | 8.47E-56 | 59.10% | C:ribosome; P:translation; F:structural constituent of ribosome; P:ribosome biogenesis | - |
|  | MDP0000220114 | mediator of rna polymerase ii transcription subunit 25-like isoform x2 | 20 | 0 | 72.40% | P:response to red or far red light; P:positive regulation of biological process; P:regulation of cellular process | - |
|  | MDP0000220174 | upf0481 protein at3g47200-like | 20 | 0 | 68.70% | - |  |
|  | MDP0000220175 | disease resistance protein rga3 | 20 | 0 | 78.10% | F:protein binding; F:ADP binding | - |
|  | MDP0000220176 | protein mitochondrial-like | 20 | 3.52E-170 | 72.15% | F:single-stranded DNA binding; P:DNA replication | - |
|  | MDP0000220177 | mitogen-activated protein kinase kinase kinase yoda-like | 20 | 0 | 76.00% | P:protein phosphorylation; F:protein tyrosine kinase activity; F:ATP binding | EC:2.7.10 |
|  | MDP0000220179 | mitogen-activated protein kinase kinase kinase yoda-like | 20 | 0 | 74.30% | P:protein phosphorylation; F:protein kinase activity; F:ATP binding | - |
|  | MDP0000220181 | gamma-tubulin complex component 5-like isoform x1 | 20 | 0 | 82.00% | P:microtubule cytoskeleton organization; C:spindle pole; C:microtubule organizing center | - |
|  | MDP0000221435 | cytochrome p450 cyp82d47-like | 20 | 0 | 81.45% | F:oxidoreductase activity, acting on paired donors, with incorporation or reduction of molecular oxygen; F:heme binding; P:oxidation-reduction process; F:iron ion binding | - |
|  | MDP0000221436 | probable transcription factor kan4 | 20 | 0 | 61.10% | F:DNA binding; F:chromatin binding; C:chromatin | - |
|  | MDP0000221442 | ent-kaurene chloroplastic-like | 20 | 0 | 88.65% | F:heme binding; F:oxidoreductase activity, acting on paired donors, with incorporation or reduction of molecular oxygen; P:oxidation-reduction process; F:iron ion binding | - |
|  | MDP0000221444 | dof zinc finger | 20 | 4.95E-148 | 69.65% | P:regulation of transcription, DNA-templated; F:DNA binding | - |
|  | MDP0000221451 | nucleobase-ascorbate transporter 3-like | 20 | 0 | 89.10% | C:membrane; F:transporter activity; P:transmembrane transport | - |
|  | MDP0000222306 | probable flavin-containing monooxygenase 1 | 20 | 0 | 86.05% | P:oxidation-reduction process; F:flavin adenine dinucleotide binding; F:N,N-dimethylaniline monooxygenase activity; F:NADP binding | EC:1.14.13.8; EC:1.14.13 |
|  | MDP0000222317 | dna mismatch repair protein msh6 | 20 | 1.65E-76 | 89.85% | P:mismatch repair; F:mismatched DNA binding; F:ATP binding | - |
|  | MDP0000224592 | | 1 | 0 | 100% | F:DNA binding; P:regulation of transcription, DNA-templated | - |
|  | MDP0000227833 | 3-ketodihydrosphingosine reductase-like | 20 | 0 | 88.50% | P:metabolic process; F:oxidoreductase activity | - |
|  | MDP0000231832 | gdt1-like protein 4 | 20 | 8.37E-69 | 89.00% | C:membrane | - |
|  | MDP0000231836 | ---NA--- | 0 |  |  | - |  |
|  | MDP0000233229 | transcription factor pif1 | 20 | 0 | 76.35% | - |  |
|  | MDP0000235023 | mediator of rna polymerase ii transcription subunit 25-like isoform x2 | 20 | 1.28E-143 | 74.45% | P:response to red or far red light; P:positive regulation of biological process; P:regulation of cellular process | - |
|  | MDP0000235369 | eukaryotic peptide chain release factor subunit 1-3 | 20 | 7.30E-100 | 67.85% | C:cytoplasm; F:translation release factor activity, codon specific; P:translational termination; F:translation release factor activity; C:cytosol; C:plasma membrane | |
|  | MDP0000239834 | allene oxide cyclase chloroplastic-like | 20 | 2.11E-140 | 85.80% | F:isomerase activity; C:chloroplast | - |
|  | MDP0000241811 | serine threonine-protein phosphatase 2a 65 kda regulatory subunit a beta isoform | 20 | 0 | 97.70% | F:binding | - |
|  | MDP0000242294 | probable plastidic glucose transporter 3 isoform x1 | 20 | 2.08E-19 | 88.70% | C:integral component of membrane; F:transmembrane transporter activity; P:transmembrane transport | - |
|  | MDP0000243706 | malonate-- ligase | 20 | 0 | 73.45% | P:metabolic process; F:catalytic activity; P:spindle assembly; C:HAUS complex | - |
|  | MDP0000244253 | copper transport protein | 20 | 7.15E-53 | 89.75% | F:metal ion binding; P:metal ion transport | - |
|  | MDP0000247199 | protein plant cadmium resistance 2-like | 20 | 6.99E-113 | 80.40% | P:pollen sperm cell differentiation | - |
|  | MDP0000247659 | peroxiredoxin chloroplastic-like | 20 | 1.31E-142 | 89.20% | P:oxidation-reduction process; F:antioxidant activity; F:oxidoreductase activity | - |
|  | MDP0000248043 | vq motif-containing protein | 20 | 2.81E-103 | 75.65% | - |  |
|  | MDP0000250967 | ctl-like protein ddb_g0274487 | 20 | 0 | 90.25% | P:regulation of transcription, DNA-templated; F:sequence-specific DNA binding transcription factor activity; F:sequence-specific DNA binding; C:transcription factor complex; P:regulation of transcription, DNA-templated | - |
|  | MDP0000251295 | 1-aminocyclopropane-1-carboxylate oxidase | 20 | 0 | 93.25% | F:oxidoreductase activity, acting on paired donors, with incorporation or reduction of molecular oxygen, 2-oxoglutarate as one donor, and incorporation of one atom each of oxygen into both donors; P:oxidation-reduction process | EC:1.14.11 |
|  | MDP0000252114 | aluminum-activated malate transporter 4 | 20 | 0 | 84.30% | P:malate transport | - |
|  | MDP0000257928 | protein glutamine dumper 5-like | 20 | 6.33E-76 | 75.65% | - |  |
|  | MDP0000257929 | cbl-interacting serine threonine-protein kinase 6-like | 20 | 0 | 83.90% | F:protein kinase activity; P:protein phosphorylation; F:ATP binding; F:omega peptidase activity | EC:3.4.19 |
|  | MDP0000257931 | 3-hydroxyisobutyryl- hydrolase-like protein mitochondrial | 20 | 0 | 82.35% | F:catalytic activity; P:metabolic process | - |
|  | MDP0000258718 | branched-chain-amino-acid aminotransferase chloroplastic-like | 20 | 0 | 83.35% | F:branched-chain-amino-acid transaminase activity; P:isoleucine catabolic process; P:leucine catabolic process; P:valine catabolic process; P:isoleucine biosynthetic process; P:leucine biosynthetic process; P:valine biosynthetic process; P:pantothenate biosynthetic process | EC:2.6.1.42 |
|  | MDP0000261040 | | 1 | 0 | 100% | F:substrate-specific transmembrane transporter activity; P:transmembrane transport; C:integral component of membrane | - |
|  | MDP0000261658 | f-box protein at1g55000-like | 20 | 4.56E-33 | 83.90% | - |  |
|  | MDP0000262602 | auxin-responsive protein iaa27-like | 20 | 0 | 77.75% | P:regulation of transcription, DNA-templated; C:nucleus; F:protein dimerization activity | - |
|  | MDP0000263035 | protein enhanced disease resistance 2-like | 20 | 0 | 88.20% | C:nucleus | - |
|  | MDP0000264232 | ---NA--- | 0 |  |  | P:oxidation-reduction process; C:membrane; F:oxidoreductase activity, acting on NAD(P)H, oxygen as acceptor; F:peroxidase activity; P:peroxidase reaction; P:response to oxidative stress | EC:1.11.1.7 |
|  | MDP0000266451 | l-type lectin-domain containing receptor kinase -like | 20 | 0 | 81.05% | P:protein phosphorylation; F:ATP binding; F:carbohydrate binding; F:protein serine/threonine kinase activity; P:serine family amino acid metabolic process | EC:2.7.11 |
|  | MDP0000266452 | uncharacterized loc101212813 | 20 | 0 | 78.20% | - |  |
|  | MDP0000266453 | | 1 | 0 | 100% | P:oxidation-reduction process; P:response to oxidative stress; F:glutathione peroxidase activity; P:RNA processing; F:RNA binding; F:nucleotide binding; P:cell cycle; P:glutathione metabolic process; P:peroxidase reaction | EC:1.11.1.9; EC:1.11.1.7 |
|  | MDP0000266454 | | 1 | 0 | 100% | F:RNA binding; F:protein binding | - |
|  | MDP0000270281 | protein fizzy-related 3 | 20 | 1.83E-50 | 90.30% | F:protein binding | - |
|  | MDP0000274967 | ---NA--- | 0 |  |  | - |  |
|  | MDP0000275365 | transmembrane fragile-x-f-associated protein | 20 | 0 | 72.85% | F:metal ion binding | - |
|  | MDP0000276832 | transposase tnp2 | 20 | 1.82E-19 | 59.00% | - |  |
|  | MDP0000286166 | dna mismatch repair protein msh6-like | 20 | 0 | 69.40% | F:mismatched DNA binding; P:mismatch repair; F:ATP binding | - |
|  | MDP0000286637 | integral membrane hpp family protein | 20 | 3.55E-127 | 82.65% | C:chloroplast inner membrane | - |
|  | MDP0000291732 | ---NA--- | 0 |  |  | - |  |
|  | MDP0000297646 | ethylene-responsive transcription factor erf003-like | 20 | 2.73E-103 | 84.50% | F:sequence-specific DNA binding transcription factor activity; P:regulation of transcription, DNA-templated; F:DNA binding; C:transcription factor complex; P:regulation of transcription, DNA-templated | - |
|  | MDP0000298053 | leucine-rich repeat receptor-like serine threonine-protein kinase at2g14440 | 20 | 0 | 83.85% | C:endosome; C:vacuole; C:trans-Golgi network; C:plasma membrane; F:kinase activity; P:phosphorylation | - |
|  | MDP0000300090 | haus augmin-like complex subunit 2 | 20 | 0 | 91.60% | F:substrate-specific transmembrane transporter activity; P:transmembrane transport; C:integral component of membrane; P:spindle assembly; P:microtubule organizing center organization | - |
|  | MDP0000300939 | septum site-determining protein mind chloroplastic | 20 | 1.16E-119 | 74.00% | C:chloroplast part; F:nucleotide binding; F:ATPase activity; F:identical protein binding; P:cellular component organization; P:single-organism cellular process | EC:3.6.1; EC:3.6.1.3; EC:3.6.1.15 |
|  | MDP0000300940 | ---NA--- | 0 |  |  | - |  |
|  | MDP0000302718 | PREDICTED: uncharacterized protein LOC103417303 | 1 | 5.37E-22 | 100.00% | - |  |
|  | MDP0000303379 | PREDICTED: uncharacterized protein LOC103416007 | 20 | 7.97E-99 | 60.30% | - |  |
|  | MDP0000303483 | protein transparent testa 1-like | 20 | 0 | 82.30% | F:nucleic acid binding; F:metal ion binding | - |
|  | MDP0000303496 | tmv resistance protein n-like | 20 | 0 | 72.30% | P:signal transduction; F:protein binding; F:ADP binding | - |
|  | MDP0000311232 | 14-3-3-like protein gf14 kappa isoform x1 | 20 | 2.80E-29 | 87.15% | F:protein domain specific binding | - |
|  | MDP0000311636 | disease resistance protein rga3 | 20 | 2.16E-21 | 71.45% | C:membrane; F:transporter activity; P:transmembrane transport | - |
|  | MDP0000312598 | | 1 | 0 | 100% | F:DNA binding; F:catalytic activity; F:pyridoxal phosphate binding | - |
|  | MDP0000312601 | lysine histidine transporter-like 8 | 20 | 0 | 97.70% | C:integral component of membrane | - |
|  | MDP0000312602 | ent-kaurene chloroplastic-like | 20 | 0 | 79.65% | P:oxidation-reduction process; F:heme binding; F:oxidoreductase activity, acting on paired donors, with incorporation or reduction of molecular oxygen; F:iron ion binding | - |
|  | MDP0000312604 | serpin-zx-like | 20 | 0 | 92.70% | C:extracellular space | - |
|  | MDP0000312606 | disease resistance protein rga3 | 20 | 0 | 79.40% | F:protein binding; F:ADP binding | - |
|  | MDP0000315002 | dof zinc finger | 20 | 1.31E-96 | 75.65% | F:DNA binding; P:regulation of transcription, DNA-templated; C:eukaryotic translation elongation factor 1 complex; F:translation elongation factor activity; C:ribosome; P:regulation of translational elongation | - |
|  | MDP0000319726 | transmembrane fragile-x-f-associated protein | 20 | 0 | 83.60% | F:protein dimerization activity | - |
|  | MDP0000321326 | glycosyl hydrolase family 17 family protein | 20 | 0 | 87.90% | P:carbohydrate metabolic process; F:hydrolase activity, hydrolyzing O-glycosyl compounds | - |
|  | MDP0000328545 | ---NA--- | 0 |  |  | - |  |
|  | MDP0000332721 | ---NA--- | 0 |  |  | - |  |
|  | MDP0000334761 | palmitoyl-monogalactosyldiacylglycerol delta-7 chloroplastic-like | 20 | 1.79E-79 | 88.80% | P:lipid metabolic process | - |
|  | MDP0000337690 | 60s ribosomal protein l39-1 | 20 | 1.73E-29 | 97.60% | C:cytosolic large ribosomal subunit; F:structural constituent of ribosome; P:translation; P:ribosome biogenesis | - |
|  | MDP0000340082 | transmembrane protein 147-like | 20 | 1.30E-169 | 95.95% | C:mitochondrion; C:Golgi apparatus | - |
|  | MDP0000348117 | ---NA--- | 0 |  |  | - |  |
|  | MDP0000353444 | rna polymerase beta subunit | 20 | 1.70E-100 | 90.95% | - |  |
|  | MDP0000357895 | ---NA--- | 0 |  |  | - |  |
|  | MDP0000375685 | transcription factor rax3 | 20 | 0 | 69.25% | F:chromatin binding; F:DNA binding; C:chromatin | - |
|  | MDP0000376284 | leucoanthocyanidin reductase-like | 20 | 0 | 91.50% | - |  |
|  | MDP0000376285 | leucoanthocyanidin reductase | 20 | 1.97E-22 | 83.60% | F:leucoanthocyanidin reductase activity; P:oxidation-reduction process | EC:1.17.1; EC:1.17.1.3 |
|  | MDP0000385620 | ribosomal protein l12 atp-dependent clp protease adaptor protein family protein isoform 1 | 20 | 2.45E-111 | 90.20% | P:protein catabolic process | - |
|  | MDP0000385622 | 40s ribosomal protein s3a-like | 20 | 1.87E-52 | 75.60% | P:translation; F:structural constituent of ribosome; C:ribosome; P:ribosome biogenesis | - |
|  | MDP0000409515 | l-type lectin-domain containing receptor kinase -like | 20 | 0 | 72.20% | F:carbohydrate binding; F:protein kinase activity; P:protein phosphorylation; F:ATP binding | - |
|  | MDP0000422612 | serine threonine-protein kinase chloroplastic | 20 | 1.65E-31 | 75.70% | F:protein serine/threonine kinase activity; F:ATP binding; P:protein phosphorylation; P:serine family amino acid metabolic process | EC:2.7.11 |
|  | MDP0000422617 | pax3- and pax7-binding protein 1 | 20 | 0 | 81.80% | P:regulation of transcription, DNA-templated; F:DNA binding; C:nucleus; F:sequence-specific DNA binding transcription factor activity; C:transcription factor complex; P:regulation of transcription, DNA-templated | - |
|  | MDP0000425402 | o-glycosyl hydrolases family 17 isoform partial | 20 | 0 | 79.10% | - |  |
|  | MDP0000445291 | clathrin assembly family protein | 20 | 0 | 71.10% | F:1-phosphatidylinositol binding; F:clathrin binding; P:clathrin coat assembly; C:clathrin-coated vesicle | - |
|  | MDP0000487985 | u2 snrnp-associated surp motif-containing isoform x1 | 20 | 4.06E-171 | 88.00% | F:nucleotide binding; F:RNA binding; P:RNA processing | - |
|  | MDP0000506508 | small ubiquitin-related modifier 1-like | 20 | 7.87E-66 | 96.40% | F:protein binding | - |
|  | MDP0000523718 | 60s ribosomal protein l13-3-like | 20 | 2.21E-27 | 77.90% | C:ribosome; P:translation; F:structural constituent of ribosome; P:ribosome biogenesis | - |
|  | MDP0000525232 | probable protein phosphatase 2c 59 | 20 | 9.78E-161 | 86.50% | F:catalytic activity | - |
|  | MDP0000559531 | septum site-determining protein mind chloroplastic | 20 | 9.16E-26 | 80.55% | C:chloroplast stroma; C:chloroplast envelope; F:ATP binding; F:calcium-dependent ATPase activity; F:protein homodimerization activity; P:barrier septum site selection; P:ATP catabolic process; P:chloroplast fission | EC:3.6.1; EC:3.6.1.3; EC:3.6.1.15 |
|  | MDP0000559532 | septum site-determining protein mind chloroplastic | 20 | 4.77E-21 | 93.50% | C:chloroplast stroma; C:chloroplast envelope; F:calcium-dependent ATPase activity; F:protein homodimerization activity; P:barrier septum site selection; P:ATP catabolic process; P:chloroplast fission | EC:3.6.1; EC:3.6.1.3; EC:3.6.1.15 |
|  | MDP0000577455 | saur family protein | 20 | 6.80E-78 | 83.00% | - |  |
|  | MDP0000600931 | eukaryotic translation initiation factor 5a-2-like | 20 | 1.66E-18 | 89.10% | C:nucleus; F:translation initiation factor activity; F:translation elongation factor activity; F:ribosome binding; P:translational frameshifting; P:peptidyl-lysine modification to peptidyl-hypusine; P:response to wounding; P:host programmed cell death induced by symbiont; P:defense response to bacterium; P:positive regulation of translational elongation; P:positive regulation of translational termination; P:response to cadmium ion; C:ribosome; P:regulation of translational initiation | - |
|  | MDP0000603942 | leucoanthocyanidin reductase-like | 20 | 0 | 91.50% | - |  |
|  | MDP0000655848 | glutamate synthase 1 | 20 | 1.58E-24 | 86.05% | C:chloroplast stroma; F:iron ion binding; F:FMN binding; F:glutamate synthase (NADH) activity; F:flavin adenine dinucleotide binding; F:iron-sulfur cluster binding; P:glutamate biosynthetic process; P:ammonia assimilation cycle; P:response to cadmium ion; P:developmental growth; P:oxidation-reduction process; P:electron transport | EC:1.4.1.14; EC:1.4; EC:1.4.1 |
|  | MDP0000663430 | zinc finger mym-type protein 5-like | 20 | 1.93E-101 | 68.55% | F:binding | - |
|  | MDP0000703817 | two-component response regulator | 20 | 0 | 76.60% | F:chromatin binding; F:DNA binding; C:chromatin | - |
|  | MDP0000717027 | 14-3-3-like protein b | 20 | 2.77E-29 | 91.35% | F:protein domain specific binding | - |
|  | MDP0000755936 | peroxiredoxin chloroplastic-like | 20 | 1.15E-103 | 95.00% | P:oxidation-reduction process; F:antioxidant activity; F:oxidoreductase activity | - |
|  | MDP0000755938 | probable flavin-containing monooxygenase 1 | 20 | 1.81E-153 | 93.60% | P:oxidation-reduction process; F:N,N-dimethylaniline monooxygenase activity; F:NADP binding; F:flavin adenine dinucleotide binding | EC:1.14.13.8; EC:1.14.13 |
|  | MDP0000755991 | polyol transporter 1 | 20 | 3.15E-35 | 67.40% | F:transmembrane transporter activity; C:integral component of membrane; P:transmembrane transport | - |
|  | MDP0000779630 | palmitoyl-monogalactosyldiacylglycerol delta-7 chloroplastic-like | 20 | 2.72E-34 | 90.15% | C:integral component of membrane; F:oxidoreductase activity, acting on paired donors, with oxidation of a pair of donors resulting in the reduction of molecular oxygen to two molecules of water; P:fatty acid biosynthetic process; P:oxidation-reduction process | EC:1.14.19 |
|  | MDP0000794936 | probable galacturonosyltransferase-like 3 | 20 | 0 | 89.75% | F:transferase activity, transferring glycosyl groups | - |
|  | MDP0000796893 | palmitoyl-monogalactosyldiacylglycerol delta-7 chloroplastic-like | 20 | 2.72E-34 | 90.15% | C:integral component of membrane; F:oxidoreductase activity, acting on paired donors, with oxidation of a pair of donors resulting in the reduction of molecular oxygen to two molecules of water; P:fatty acid biosynthetic process; P:oxidation-reduction process | EC:1.14.19 |
|  | MDP0000806021 | palmitoyl-monogalactosyldiacylglycerol delta-7 chloroplastic-like | 20 | 6.29E-98 | 82.40% | F:oxidoreductase activity, acting on paired donors, with oxidation of a pair of donors resulting in the reduction of molecular oxygen to two molecules of water; P:oxidation-reduction process; P:lipid metabolic process | EC:1.14.19 |
|  | MDP0000816018 | palmitoyl-monogalactosyldiacylglycerol delta-7 chloroplastic-like | 20 | 3.45E-29 | 95.05% | C:integral component of membrane; F:oxidoreductase activity, acting on paired donors, with oxidation of a pair of donors resulting in the reduction of molecular oxygen to two molecules of water; P:fatty acid biosynthetic process; P:oxidation-reduction process | EC:1.14.19 |
|  | MDP0000816846 | probable inactive receptor kinase at1g48480 | 20 | 3.19E-75 | 70.25% | C:membrane; F:nucleotide binding; F:protein kinase activity; P:metabolic process | - |
|  | MDP0000822021 | adenosylhomocysteinase | 20 | 0 | 98.40% | F:adenosylhomocysteinase activity; P:one-carbon metabolic process; P:methionine metabolic process | EC:3.3.1.1; EC:3.3.1 |
|  | MDP0000836363 | ---NA--- | 0 |  |  | - |  |
|  | MDP0000859609 | probable calcium-binding protein cml18 | 20 | 2.63E-114 | 81.90% | F:calcium ion binding | - |
|  | MDP0000868486 | hypothetical protein PRUPE_ppa022986mg, partial | 4 | 6.32E-27 | 60.75% | - |  |
|  | MDP0000881783 | transmembrane protein 147-like | 20 | 1.35E-169 | 95.95% | C:mitochondrion; C:Golgi apparatus | - |
|  | MDP0000881805 | probable thylakoidal processing peptidase chloroplastic | 20 | 0 | 82.30% | F:serine-type peptidase activity; P:proteolysis; C:integral component of membrane | - |
|  | MDP0000912045 | nucleobase-ascorbate transporter 3-like | 20 | 2.81E-103 | 87.25% | F:transporter activity; C:membrane; P:transmembrane transport | - |
|  | MDP0000912056 | ---NA--- | 0 |  |  | - |  |
|  | MDP0000912059 | thioredoxin-like protein chloroplastic | 20 | 3.86E-133 | 82.65% | P:cell redox homeostasis; F:protein disulfide oxidoreductase activity; P:glycerol ether metabolic process; P:electron transport | - |
|  | MDP0000939633 | ap2 erf and b3 domain-containing transcription factor rav1 | 20 | 0 | 78.35% | P:regulation of transcription, DNA-templated; F:sequence-specific DNA binding transcription factor activity; F:DNA binding; C:transcription factor complex; P:regulation of transcription, DNA-templated | - |
|  | MDP0000941318 | bola-like protein 1 | 20 | 5.43E-117 | 76.60% | C:chloroplast | |
|  | MDP0000952005 | compass component swd1 | 20 | 6.61E-93 | 96.95% | F:protein binding | - |
|  | MDP0000952010 | kinesin-like protein kif11 | 20 | 0 | 84.70% | - |  |

^a^ Mean Similarity

^b^ Gene Ontology
